# Distribution Analyzer, a methodology for identifying and clustering outlier conditions from single-cell distributions, and its application to a *Nanog* reporter RNAi screen

**DOI:** 10.1186/s12859-015-0636-7

**Published:** 2015-07-22

**Authors:** Julian A. Gingold, Ed S. Coakley, Jie Su, Dung-Fang Lee, Zerlina Lau, Hongwei Zhou, Dan P. Felsenfeld, Christoph Schaniel, Ihor R. Lemischka

**Affiliations:** 1 The Black Family Stem Cell Institute, Icahn School of Medicine at Mount Sinai, New York, NY 10029 USA; 2 Department of Developmental and Regenerative Biology, Icahn School of Medicine at Mount Sinai, New York, NY 10029 USA; 3 Program in Applied Mathematics, Yale University, New Haven, CT 06511 USA; 4 Cancer Biology and Genetics Program, Memorial Sloan Kettering Cancer Center, New York, NY 10065 USA; 5 Integrated Screening Core, Experimental Therapeutics Institute, Icahn School of Medicine at Mount Sinai, New York, NY 10029 USA; 6 Department of Pharmacology and Systems Therapeutics, Icahn School of Medicine at Mount Sinai, New York, NY 10029 USA

**Keywords:** Genome-scale screen analysis, Fluorescence distribution, High-content screening methodology, *Nanog* RNAi screen, Hellinger distance, Kolmogorov-Smirnov distance

## Abstract

**Background:**

Chemical or small interfering (si) RNA screens measure the effects of many independent experimental conditions, each applied to a population of cells (e.g., all of the cells in a well). High-content screens permit a readout (e.g., fluorescence, luminescence, cell morphology) from each cell in the population. Most analysis approaches compare the average effect on each population, precluding identification of outliers that affect the distribution of the reporter in the population but not its average. Other approaches only measure changes to the distribution with a single parameter, precluding accurate distinction and clustering of interesting outlier distributions.

**Results:**

We describe a methodology to identify outlier conditions by considering the cell-level measurements from each condition as a sample of an underlying distribution. With appropriate selection of a distance metric, all effects can be embedded in a fixed-dimensionality Euclidean basis, facilitating identification and clustering of biologically interesting outliers. We demonstrate that measurement of distances with the Hellinger distance metric offers substantial computational efficiencies over alternative metrics. We validate this methodology using an RNA interference (RNAi) screen in mouse embryonic stem cells (ESC) with a *Nanog* reporter. The methodology clusters effects of multiple control siRNAs into their true identities better than conventional approaches describing the median cell fluorescence or the commonly used Kolmogorov-Smirnov distance between the observed fluorescence distribution and the null distribution. It identifies outlier genes with effects on the reporter distribution that would have been missed by other methods. Among them, siRNA targeting *Chek1* leads to a wider *Nanog* reporter fluorescence distribution. Similarly, siRNA targeting *Med14* or *Med27* leads to a narrower *Nanog* reporter fluorescence distribution. We confirm the roles of these three genes in regulating pluripotency by mRNA expression and alkaline phosphatase staining using independent short hairpin (sh) RNAs.

**Conclusions:**

Using our methodology, we describe each experimental condition by a probability distribution. Measuring distances between probability distributions permits a multivariate rather than univariate readout. Clustering points derived from these distances allows us to obtain greater biological insight than methods based solely on single parameters. We find several outliers from a mouse ESC RNAi screen that we confirm to be pluripotency regulators. Many of these outliers would have been missed by other analysis methods.

**Electronic supplementary material:**

The online version of this article (doi:10.1186/s12859-015-0636-7) contains supplementary material, which is available to authorized users.

## Background

High-content screening has become a popular experimental tool to study the effects of a large number of compounds or single-gene knockdown conditions on individual cells, offering a fine-grained cell-level characterization of response to a large number of treatments [1–3]. Studies that utilize high-content microscopy have become more practical thanks to the development of siRNA and chemical libraries and have provided mechanistic insights into the regulation of complex phenotypes [4]. Embryonic stem cells (ESCs) are among the most popular of the systems studied with high-content screening in the search for regulators of pluripotency and differentiation. In these studies, fluorescent reporters are often driven by pluripotency genes such as *Pou5f1* (gene id 18999) [5–10], *Nanog* (gene id 71950) [11–13] and *Zfp42* (gene id 22702, also known as *Rex1*) [14, 15].

Transcriptional heterogeneity in ESCs has been demonstrated to regulate pluripotency and cell fate decisions [16–21]. An analysis of the regulators of heterogeneity of ESC populations is thus of substantial scientific interest. However published ESC screens [5–15, 22, 23] have yet to exploit the cellular-level data in their analyses, although others have utilized variants of the Kolmogorov-Smirnov (KS) statistic to compare single-cell distributions [24–27]. While such screens theoretically permit the study of an entire population of treated cells, most currently-applied computational methods, including those that analyze the cell-level distribution with the KS statistic, reduce the effect of a treatment to a single (univariate) parameter, such as median or mean cell fluorescence [28–32] or KS distance of the fluorescence distribution to the null effect distribution [24–27]. In doing so, a significant amount of potentially informative data about the individual cells, each with distinct levels of reporter expression, is typically disregarded. For example, effects leading to more uni- or bi-modal, or to narrower or broader, fluorescence distributions, may not affect the mean or median fluorescence but may be of substantial biological interest. As we demonstrate below, such situations regularly arise in the context of high-content screening and convey biologically relevant information. Therefore, a generalizable method for screens that can extract a multivariate readout from the univariate single-cell distributions is desired.

Here, we describe the types of screens amenable to such distribution-based analysis, provide a novel and broadly applicable approach and describe the needed steps to reduce terabytes of high-resolution images of treated cells to a small number of the most relevant and interpretable parameters measuring the effects. We demonstrate its validity when applied to the raw data of our previously-described small interfering (si) RNA screen using a fluorescent *Nanog* pluripotency reporter mouse (m) ESC line [12]. Using our approach we are able to a) reliably distinguish between conditions whose effects appear comparable when scored using conventional methodologies, b) identify outliers in the screen using a specified Z-score cutoff and c) classify outliers based on changes to their cell-level fluorescence distributions, assigning them to prototypical outlier effect categories. In the process, we identify a number of novel regulators of pluripotency that would have been missed by conventional methodologies.

### Methodology

A distribution-based methodology can be applied to analyze high-content screens in which the effect from each experimental condition (e.g., a well treated with a particular siRNA or chemical) is measured at the single-cell level. These measurements are typically made when a collection of cells within a well of a screening plate is imaged. Specialized software packages process the images to extract parameter(s) for each cell, e.g., average fluorescence per cytoplasmic pixel. Cellular-level data is also routinely measured in screens using a flow cytometer that detects fluorescence and/or scatter. The methodology described below is for univariate cell-level input data (when each cell is described with one parameter). It provides a multivariate condition-level (or well-level) output.

**Fig. 1:**
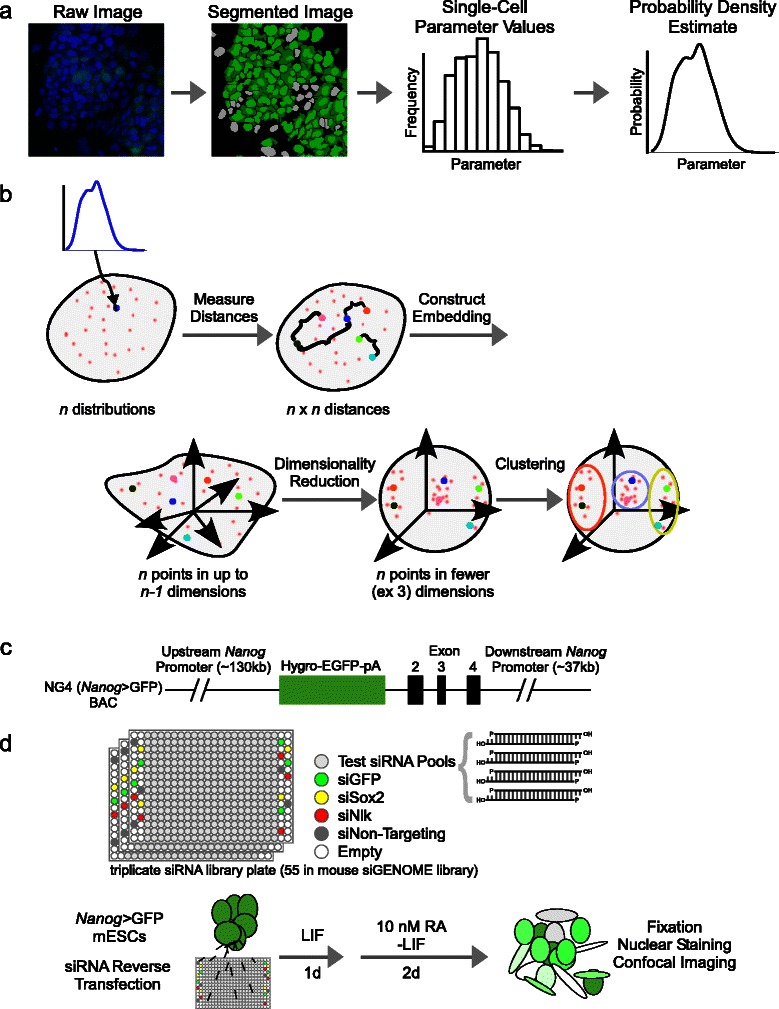
Workflow for distribution-based methodology. **a** Processing of raw images into distributions. Images are segmented based on nuclear staining (blue) and cytoplasmic GFP (green) to yield cytoplasmic fluorescence intensities for each cell (green or grey, if below background). These values are used to estimate a probability distribution for the parameter. **b** Schematic of single-cell distribution-based methodology. Parameter values are converted into a probability distribution estimate. The distances between each probability distribution are used to assign each condition a point in Euclidean space. Dimensionality reduction is performed using PCA and clustering applied to distinguish effects and categorize the outliers. **c** NG4 line vector [11]. The BAC-based GFP reporter is driven by the *Nanog* promoter. **d** Schematic of siRNA screen as previously described [12]. Pools of siRNA covering the mouse genome are printed onto 55 384-well plates along with controls in triplicate. NG4 cells are reverse transfected and cultured for 3 days under mild differentiation conditions. Cells are fixed, nuclei stained and plates imaged at cell-level resolution

The distribution-based methodology consists of the following steps as summarized in Fig. 1a, b. R source code for the described methodology and analysis, including sample data, can be found in Additional file 1: Code S1.

### Normalization

Because large-scale screening is necessarily staggered across multiple data acquisition sets (e.g., into 384-well screening plates or flow cytometer runs, each of which has its own set of controls), normalization is a prerequisite for comparing samples from different sets. Cell culture technique, post-fixation handling and microscopy all contribute to technical variability.

Normalization ensures, for example, that a cell highly fluorescent relative to the other cells on plate A is treated the same way as a highly fluorescent cell on plate B. Similarly, it ensures that non-fluorescing cells on plate A have approximately the same background fluorescence level as non-fluorescing cells on plate B.

Parametric affine transformation (i.e., translation plus a linear scale) is the conventional normalization method used in the screening field [31]. Affine transformations include all of the normalization methods implemented in the popular screen analysis R package cellHTS2 [31], including control-based normalization (e.g., percent of control, normalized percent inhibition) and non-control based normalization (e.g., plate median normalization, Z-score, B-score) methods.

However, the methodology identifies certain probability distribution functions as outliers in a non-parametric space of probability distribution functions. Its normalization should ensure that the distribution distance (described below) between probability distributions of identical conditions across datasets (plates) is as close to zero as possible. An affine transformation (whether by control or non-control methods) offers no such guarantee. Therefore, affine transformation is not generally recommended.

Instead, an initial non-parametric normalization is required to compensate for the technical variation between screening plates. Because the probability distributions derive from individual cell measurements, the non-parametric normalization is determined from and applied to cell-specific values rather than well-specific values.

While an affine transformation is entirely defined by two values (e.g., mean and standard deviation), a non-parametric normalization is specified by a collection of values. We determine these values from a reference dataset (plate). Any representative plate from the screen that has passed quality control validation (see Screen Quality Control in Results) is an appropriate choice for reference plate.

The non-parametric normalization can be non-control based or control-based. Prior to normalization across plates, parameter values, e.g., fluorescence, for each cell are transformed to a logarithmic scale.

#### Non-control based non-parametric normalization

If cell populations across datasets (e.g., all of the cells on a screening plate) can be considered comparable (typically, because the controls on each screening plate are fixed and the vast majority of the non-control experimental conditions have no effect), non-control based non-parametric normalization can be employed. Given *w* cells on a reference plate, whose fluorescence is ranked from 1 to *w*, and *v* cells on the plate to be normalized, whose fluorescence is ranked from 1 to *v*, the fluorescence of the i^th^ ranked cell on the plate to be normalized is assigned the value of the ${\left(\left(w-1\right)\frac{i-1}{v-1}+1\right)}^{th}$ ranked cell in the reference dataset, with linear interpolation.

For example, if there are *w* = 1,200,001 cells in the 384-well reference plate and *v* = 1,000,001 cells in the plate to be normalized, the fluorescence of the cell with the 750,001^th^ highest ranked fluorescence on the plate to be normalized will be reassigned to the fluorescence of the ${\left(1,200,000\frac{750,000}{1,000,000}+1\right)}^{th}=900,{001}^{th}$ highest ranked cell in the reference plate. Normalization effects on plates of siRNA libraries for our *Nanog*-GFP reporter line following non-control based non-parametric transformation are shown in Additional file 2: Figures S1a, S1b, Additional file 3: Figure S2c and Additional file 4: Figure S3.

#### Control-based non-parametric normalization

If only the controls across datasets (plates) can be considered comparable (e.g., if the non-control wells on one plate are expected to have consistently different distributions than the non-control wells on another plate), a control-based non-parametric normalization is required.

Given *b* different types of controls present on every plate, labeled i_1_, i_2_, … i_b_ (e.g., *b* = 5 types of controls on each plate named siGFP, siSox2, siNon-targeting, Empty, and siNlk), and n(i_j_) cells present in the wells on the plate treated with control i_j_, a reference plate of controls is generated specific to a given plate to be normalized. The cell (fluorescence) values from the reference plate for each control condition i_j_ are sampled with replacement n(i_j_) times, where n(i_j_) is the number of cells of type *i*_j_ on the plate to be normalized. The sampling procedure is performed by pseudorandom number generation with a fixed seed. This procedure addresses the variability in the number of cells (and wells) of a given control type across datasets.

Thus the number of control cells on the generated reference plate is guaranteed to be identical to the number of control cells on the plate to be normalized, namely ${\displaystyle \sum_{j=1}^b}n\left({i}_j\right)$. The sorted values from the plate to be normalized are mapped to the sorted values of the generated reference plate. The values of all other cells not considered controls on the plate to be normalized are derived by linear interpolation from this mapping. Any values outside the interval of control values are assigned the value of the closest data extreme.

For example, consider a plate with two types of controls, A and B, that is to be normalized to a reference plate. The values of cells on the reference plate corresponding to i_1_ (“control A”) are (5,2,4) and the values of cells on the reference plate corresponding to i_2_ (“control B”) are (6,4,8,7). The values of cells on the plate to be normalized corresponding to control A are (4,3,5,4) and the values of cells on the reference plate corresponding to control B are (8,7,5). Note that the number of cells of each control type on the two plates may differ. The values from the reference plate are sampled 4 times from control A and 3 times from control B, yielding (in one outcome of a random sampling) (2,5,4,5) for control A and (7,4,7) for control B. All these control values on the original and simulated reference plate are aggregated and sorted to define the mapping from which interpolation is performed: (3,4,4,5,5,7,8) → (2,4,4,5,5,7,7). By this mapping, non-control cells with the values (1,3.5,5,8,6,10) are interpolated to their new normalized values of (2,3,5,7,6,7).

Control-based non-parametric normalization is limited by the smaller number of cells on a dataset that can be used for normalization versus non-control based methods, possibly resulting in a coarser interpolation.

An example of control-based non-parametric transformation is shown in Additional file 3: Figure S2b.

#### Comparison of normalization methods

The effects of two plates following parametric normalization by Z-score [31], control-based non-parametric normalization and non-control based non-parametric normalization were compared (Additional file 3: Figure S2). The distributions between control wells closely lined up across plates following normalization by all methods, including the theoretically inappropriate Z-score normalization, suggesting that many approaches to normalization are likely valid.

### Construct a distribution for each condition by kernel density estimation

For each condition, the readout from single cells is used to construct a smooth estimate of the probability density function (PDF, also called the probability distribution) by Gaussian kernel density estimation [33]. The probability density function is estimated over the domain [a,b], defined by the domain of the reference plate cell-level values. Non-parametric normalization (above) guarantees that cell values from every plate fall within this domain.

Each probability distribution *p(x)* on [a,b] may be approximately represented as a discrete probability distribution *p(x*_*i*_ *)* = *(p(x*_*1*_ *),…,p(x*_*m*_ *))* on a set of *m* bins whose bin centers are *(x*_*1*_ *,…,x*_*m*_ *)*, where *x*_*1*_ = a and *x*_*m*_ = b. The width of the bins is determined by the bandwidth of the kernel density estimation, chosen to be 0.9 times the minimum of the standard deviation and the inter-quartile range divided by 1.34 times the sample size to the negative one-fifth power [33]. The discrete probability distribution is scaled such that ${\displaystyle \sum_{i=1}^m}p\left({x}_i\right)=1$.

A sufficient number of cells must be present in each condition in order to estimate the true probability distribution with reasonable confidence. A *minimumcells* parameter is chosen so that the error in generation of the distributions remains smaller than the variation between known null-effect conditions. We disregard conditions in which fewer than *minimumcells* cells are identified following image processing (e.g., conditions that lead to substantial cell death) to avoid constructing unreliable estimates of the fluorescence distribution.

In our screen dataset, the KS statistic between the probability distribution estimate and the actual cell measurements was consistently greater than 0.05 in wells with fewer than 100 cells when *m* = 512 (Additional file 5: Figure S4). In contrast, the median KS statistic between empty wells and the screen-averaged null-effect was 0.087. Therefore, the *minimumcells* parameter for density estimation in DistributionAnalyzer was set to a default value of 100 because generation of a probability distribution from wells with >100 cells in our dataset added an amount of error that is less than existing experimental variation. A larger value of *minimumcells* may also be selected by the user for biological reasons. The software can optionally disregard conditions in which the error in estimating the probability exceeds a given KS, whose default is 0.05.

The number *m* of equally spaced bins for density estimation is a user-definable parameter in DistributionAnalyzer with a default of 512. The error in estimation of distributions from individual cell measurements, as measured by the KS statistic, is essentially unchanged for *m* = 256, 512 and 1024 (Additional file 6: Figure S5), particularly for larger *m*, as the bandwidth in kernel density estimation does not depend on *m*.

In practice, these requirements do not present a significant challenge to conducting a screen. A 384-well plate can (and typically will) contain several thousand cells per well. In our screen, 99.4 % of all (non-control) wells had >100 cells, 98.9 % had a KS error <0.05 when *m* = 512, and 98.8 % met both criteria. Most of the screened wells that did not meet these criteria were controls, such as siWee1, chosen to induce cell death (data not shown).

The series of transformations resulting in a construction of a probability distribution is depicted in Fig. 1a and Additional file 2: Figure S1c.

### Measure distances between distributions by selection of a distance metric

A distance metric is used to compute a dissimilarity score between two distributions. There are many appropriate choices of distance metric. Any standard or classical metric defined on the full (Fréchet) space of one-dimensional probability distributions on an interval is available [34].

For example, the two-sample KS statistic (distance) has been widely used in the screening literature to measure distances between distributions [24–27] and is defined as $\mathrm{K}\mathrm{S}\left(p\left({x}_i\right),q\left({x}_i\right)\right)=\underset{y}{ \max}\left|{\displaystyle \sum_{x_i\le y}}p\left({x}_i\right)-{\displaystyle \sum_{x_i\le y}}q\left({x}_i\right)\right|$. The KS statistic is only one of many available statistics suitable for measuring the “distance” of an empirical distribution function to a reference distribution function (a “goodness of fit” test). The Anderson-Darling statistic, the Ryan-Joiner statistic, and the Chi-Squared statistic, for example, are arguably even more popular choices [35–37], as are the Lévy distance (Lévy-Prokhorov metric), Wasserstein (earth-mover) distance and Hellinger distance [38–41].

Moreover, any one of these statistics, as well as any “f – divergence” (including Chi-squared divergence, Kullback–Leibler divergence, total-variation divergence, Jenson-Shannon divergence, alpha divergence, etc.) can be used (by symmetrization) to define at least one distance metric on empirical distribution functions.

Among the many established distance metrics to calculate the distance between probability distributions, we select the *Hellinger distance* [41, 42], which is defined for two discretized probability distributions *p(x*_*i*_ *)* and *q(x*_*i*_ *)* on an interval [a,b] as $\mathrm{H}\mathrm{D}\left(p\left({x}_i\right),q\left({x}_i\right)\right)=\sqrt{{\displaystyle \sum_{i=1}^m}{\left(\sqrt{p\left({x}_i\right)}-\sqrt{q\left({x}_i\right)}\right)}^2}$ and ranges from 0 to √2. The selection of the Hellinger distance offers substantial advantages in computational complexity as well as in clustering accuracy and interpretation (see Results).

The comparative study of metric choices is in generality more appropriately treated in mathematical (information geometry) literature.

### Represent each condition effect as a point in high-dimensional Euclidean space

We construct an *isometric embedding* [43] (mapping) such that each condition is represented as a point in Euclidean space whose Euclidean distance to any other condition is the metric distance between the corresponding distributions (Fig. 1b, left).

#### For an arbitrary choice of distance metric

Determining such an *isometric embedding* for *n* points from a *statistical manifold* (the space in which the distributions reside) [43] is always possible by the Nash embedding theorem with a Euclidean space of dimension at most *n-1*, for example by the method of *multidimensional scaling* (MDS) [44]. Thus, regardless of the metric choice, we can always represent the *n* distributions as a set (of size *n*) of *n-1* dimensional points. Computationally, the procedure of MDS up to an arbitrary dimensionality (up to *n-1*) can be performed using the “cmdscale” command in R. This sequence is depicted in Fig. 1b.

The net effect of this procedure will be to represent each distribution *p(x)* in the set of *n* distributions as a point *(r*_*1*_ *, r*_*2*_ *,…,r*_*n-1*_ *).*

#### For the choice of the Hellinger distance metric

The computationally intensive task of MDS on all pairwise distances can be sidestepped entirely with the selection of the Hellinger distance.

Consider the maps $\left({r}_1,\dots, {r}_m\right)=\left(\sqrt{p\left({x}_1\right)},\dots, \sqrt{p\left({x}_m\right)}\right)$ and $\left({s}_1,\dots, {s}_m\right)=\left(\sqrt{q\left({x}_1\right)},\dots, \sqrt{q\left({x}_m\right)}\right)$.

The Hellinger distance between two discretized probability distributions *p(x*_*i*_ *)* and *q(x*_*i*_ *)* can be simplified as

$$ \begin{array}{ccc}\hfill \hfill & \hfill \mathrm{H}\mathrm{D}\left(p\left({x}_i\right),q\left({x}_i\right)\right)\kern1em =\hfill & \hfill \sqrt{{\displaystyle \sum_{i=1}^m}{\left(\sqrt{p\left({x}_i\right)}-\sqrt{q\left({x}_i\right)}\right)}^2}\hfill \\ {}\hfill \hfill & \hfill =\hfill & \hfill \sqrt{{\displaystyle \sum_{i=1}^m}{\left({r}_i-{s}_i\right)}^2}.\hfill \end{array} $$

The vector *(r*_*1*_ *,…,r*_*m*_ *)* is thus an isometric embedding as sought, as the pair-wise Euclidean distance between any *(r*_*1*_ *,…,r*_*m*_ *)* and *(s*_*1*_ *,…,s*_*m*_ *)* is the pair-wise Hellinger distance between the discretized distributions *p(x*_*i*_ *)* and *q(x*_*i*_ *)*. In this manner, the computationally expensive construction of an isometric embedding (e.g., by classical MDS), can be avoided. Moreover, as this embedding for the Hellinger distance is of dimensionality *m* rather than the typically much larger dimensionality *n-1*, determined by the number of experimental conditions, dimensionality reduction (below) by Principal Component (PC) Analysis (PCA) is far more computationally tractable.

Note that the Euclidean embedding *(r*_*1*_ *,…,r*_*m*_ *)* is simply the square root of the discrete probability distribution.

### Singular value decomposition

The condition effects on the set of single-cell distributions are represented as a *n x* *p* matrix Q, constructed from the *n* *p*-dimensional points (the embedding). For the Hellinger distance metric, *p* = *m*.﻿ For all other distance metrics, *p* = *n*-1. The matrix Q represents the set of effects in a Euclidean space. We perform PCA on Q by computation of the (truncated) singular value decomposition (SVD) (Fig. 1b, right). This is performed with the “svd” function in R.

For example, if each kernel density estimate is represented as values at *m* = 512 discrete points and there are 20,000 conditions screened in technical triplicate along with 5,000 other control sites,  *n*=65,000. The isometric embedding using the Hellinger distance is also of dimensionality *p* =*m* and a 65,000 X 512 matrix is obtained. For an arbitrary distance metric, this matrix will be 65,000 x 64,999.

The SVD of *Q* breaks down each condition’s effect (the matrix row $\overrightarrow{Q_i}$) into a linear combination of right singular vectors (PC_j_ effects), scaled by the corresponding left singular vector weightings $\overrightarrow{w_i\;}$, such that $\overrightarrow{Q_i}={w}_{i1}\overrightarrow{P{C}_1}+{w}_{i2}\overrightarrow{P{C}_2}+\cdots +{w}_{ip}\overrightarrow{P{C}_p}$, with $\sum_{j=1}^p{w_{ij}}^2=1$.

#### Features of the SVD when using the Hellinger distance embedding

Because the Hellinger distance embedding is defined as the square root of the discrete probability distribution (see above), the SVD of *Q* built from the Hellinger distance breaks down each “square root distribution” (represented as a row vector $\overrightarrow{Q_i}$) into a linear combination of eigen-“square root distributions.”

**Fig. 2:**
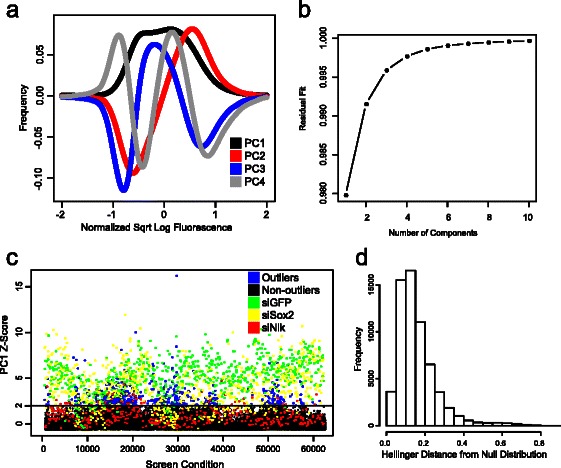
Applying the distribution-based methodology to *Nanog* siRNA screening data. **a** The first 4 PCs (right singular vectors) from the application of the methodology using the Hellinger distance metric to the screen data, noted as PC1, PC2, PC3 and PC4. The fluorescence distribution of each condition (well) is approximately the square of a linear combination of these PCs. **b** Cumulative square root residual sum of squares plot for first 10 PCs. Nearly all the screen information is captured using few PCs. **c** Plot of the PC1 values across the genome-wide screen. Conditions with |Z-score| >2 in PC1 that differ strongly from the overall distribution are shown in blue. siGFP is shown in green, siSox2 in yellow and siNlk in red. All other conditions are shown in black. **d** Histogram of Hellinger distance of all probability distributions to the mean probability distribution over all screen sites

The first right singular vector (shown in black in Fig. 2a) is the average of all such “square root distributions.” Because almost all conditions are expected to have no effect, it can be thought to represent the “overall” null parameter effect across all samples in the screen. Because ${\displaystyle \sum_{j=1}^m}{w_{ij}}^2=1$, conditions that most closely overlap with the null “square root distribution” (*|w*_*i1*_ *|* closest to 1) will have nearly zero weight in their remaining PCs. Conditions with smaller *|w*_*i1*_ *|* values have poorer fits to the null distribution and represent the most distinct “square root distributions,” comprised in greater part of effects along the higher-order right singular vectors.

The higher-order right singular vectors represent the greatest sources, in order, of modulations to the null effect. While the weights for the first right singular vector are all of the same sign (conventionally positive), the weights of the higher-order right singular vectors can be either positive or negative. Although a biological interpretation of the right singular vectors is, in general, not guaranteed, the second right singular vector (red in Fig. 2a) may represent an “up or down” effect in overall fluorescence, while the third right singular vector (blue in Fig. 2a) may represent an effect on reporter variance.

### Dimensionality reduction

An appropriate number *k* of PCs is chosen so that only the PCs that explain more than a minimum threshold of residual fit to the true distributions (Fig. 1b) are saved. The remaining PCs are considered experimental noise and discarded. This residual fit threshold is chosen arbitrarily to be 0.001 (i.e., one thousandth of the variation in the data). However, because the utility of a methodology designed to produce a multivariate output from univariate distributions would be limited for *k* < 3, the minimum value for *k* is set to 3.

With the residual fit threshold described above, we found *k* = 4 PCs for our screen, although application to other screen datasets often yielded *k* = 3 (data not shown). The *k* left singular vectors (as an *n* by *k* matrix) scaled by the *k* corresponding singular values, may be interpreted as *n* points in *k*-dimensional space.

The null effect (and its corresponding null distribution) can either be represented as a) the *k*-dimensional point at the scaled center (mean) of all conditions, if most conditions can be assumed to have no effect, as was the case in our screen, or b) the *k*-dimensional point at the center of the points representing the null-effect or non-targeting controls, also scaled to unit magnitude.

Each *k*-dimensional point *(w*_*i1*_ *, w*_*i2*_ *,…, w*_*ik*_ *)* can be converted back into an approximation of the embedding, called $\overrightarrow{\overset{\sim }{Q_i}}$, where $\overrightarrow{Q_i}\approx \overrightarrow{\overset{\sim }{Q_i}}={w}_{i1}\overrightarrow{P{C}_1}+{w}_{i2}\overrightarrow{P{C}_2}+\dots +{w}_{ik}\overrightarrow{P{C}_k}$.

#### Features of the dimensionality reduction when using the Hellinger distance embedding

The magnitude of the vector *(r*_*1*_ *,…,r*_*m*_ *)* can be computed as $\parallel {r}_1,\dots, {r}_m\parallel =\sum_{i=1}^m{r}_i^2=\sum_{i=1}^mp\left({x}_i\right)=1$. Therefore each *(r*_*1*_ *,…,r*_*m*_ *)* has unit magnitude and is a point on the surface of an *m*-dimensional sphere of unit radius. The *k*-dimensional row vectors from the SVD that approximate each vector *(r*_*1*_ *,…,r*_*m*_ *)* also have nearly unit magnitude. They thus represent points that fall very near the surface of a *k*-dimensional sphere of unit radius. Because the Hellinger distance embedding is an invertible map from the discretized distributions, each point can also be converted back into a probability distribution as $\overrightarrow{{\overset{\sim }{Q_i}}^2}$.

### Outlier identification

The Euclidean distance of each condition from the null effect (however it is determined) represents how much each condition changes the distribution. These distances are rescaled and treated as Z-scores, with an adjustable significance cutoff, typically |Z-score| >2. Outliers are arbitrarily defined as conditions whose median |Z-score| >2 over at least 2 technical replicates. All condition effects with median |Z-score| ≤2 are considered to be non-significant. A Z-score criterion is applied because most conditions have no effect and distances between null effect condition distributions to the null effect mean distribution are nearly normally distributed (Fig. 2d and Additional file 7: Table S1).

Acceptable alternative outlier identification criteria exist [6, 7, 24–32, 45–47] but are not discussed here.

### Outlier categorization

The conditions with significantly changed (outlier) distributions, each of which is represented as a *k*-dimensional point, are categorized using partitioning around medoids clustering (Fig. 1b, right) with a specified number of clusters. The number of clusters may be determined by several means:

- A cluster number that maximizes the silhouette width criterion to divide the screen outliers into their most “natural” compact grouping.

**Fig. 3:**
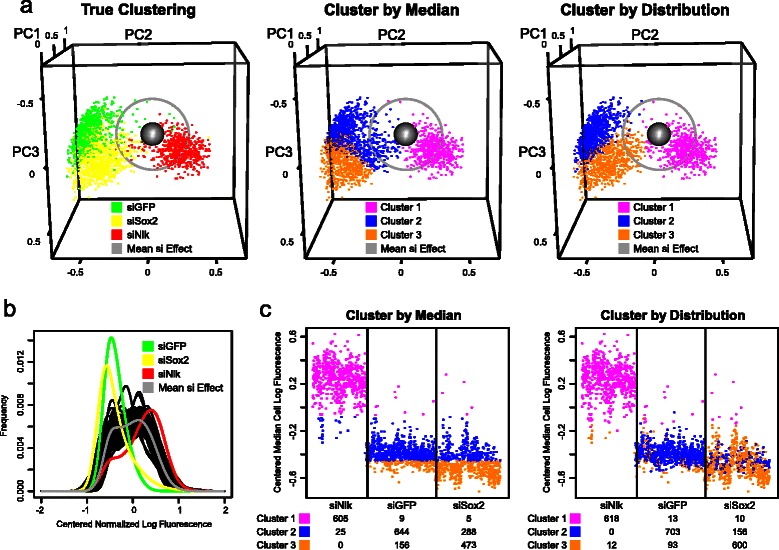
Distinguishing between siRNA conditions using median cell- and distribution-based clustering. **a** The weighting along the first three PCs for each siNlk, siGFP and siSox2 condition (well) with >100 cells is plotted as a point in three dimensions. The mean (presumed null) effect across all conditions is shown as a grey sphere. Grey circle defines all conditions within a 2 Z-score Hellinger distance from the mean effect. Left: siNlk, siGFP and siSox2 wells are colored in red, green and yellow, respectively. Center: Color assignment is determined by 3 medoid clustering of the median cell fluorescence. Right: Color assignment is determined by 3 medoid clustering of the first 4 PCs weights. **b** Mean fluorescence distribution for siGFP, siSox2 and siNlk conditions (green, yellow and red, respectively) and mean distribution across all conditions in siRNA screen (grey). Distribution mean is derived from average over all replicates in distribution score (PC) space. The distributions from several representative null-effect wells are shown in black. **c** Median cell fluorescence for each control siRNA condition (well), ordered by category (siNlk, siGFP and siSox2). Colors represent 3 medoid clustering either by median cell fluorescence (left) or by distribution (right), as in Fig. 3a. Values beneath each siRNA represent number of conditions assigned to each cluster
- A cluster number greater than 2 that maximizes the silhouette width criterion to divide the screen outliers more finely than using an “up-or-down” grouping.
- A user-specified cluster number to divide the screen outliers into a known number of states defined by a biological mechanism.

## Results

We applied our methodology to the processed cell-level data from our previously described genome-wide RNAi screen [12]. This screen used the NG4 mESC *Nanog*-GFP pluripotency reporter line [5, 11] cultured under mild, retinoic acid (RA)-induced differentiation conditions (Fig. 1c). GFP fluorescence is produced under control of the *Nanog* promoter region and is expected to correlate with pluripotency. mESCs were transfected with siRNA pools in 384-well plates, cultured in LIF-containing media for 1 day and grown for 2 additional days without LIF and with 10 nM RA (Fig. 1d). Confocal fluorescent microscopy on the fixed, Hoechst-33342 nucleus-stained cells provided images with cell-level resolution for each condition in the ThermoScientific siGENOME library targeting 16872 mouse genes along with assay-specific controls in technical triplicate. Under these conditions siRNA pools that decrease reporter fluorescence as well as those that increase it can be identified. Therefore, positive as well as negative effects on the pluripotency loss can be identified in one screen.

We applied our methodology to measure effects of each condition on the distribution as measured by the Hellinger distance. We computed the Euclidean embedding of Hellinger distances in our screen and performed a SVD on the embedding to identify the PC scores (Fig. 2ab). We found that a *k* = 4 dimensional best fit subspace was more than sufficient to capture the screen effects (Fig. 2b). The relative residual sum of squares (RRSS) for the 4-dimensional projection was >99.5 % and the second through fourth dimensions captured >94 % of the RRSS not already captured by the first (i.e., the residuals from second, third, …, *m* = 512th).

### Screen quality control

The well-to-well variation from the empty and siGFP controls in our screen was assessed. The *t*-test between the set of Hellinger distances of GFP-reducing controls to null effect and the Hellinger distances of empty wells to the null effect was computed for each plate (Additional file 4: Figure S3). Most p-values were on the order of 10^−4^ or smaller, reflecting a strong discrimination between empty wells and GFP-reducing controls.

The plate-to-plate variation from the empty and siGFP controls in our screen was assessed (Additional file 4: Figure S3). The standard deviation of Hellinger distance of empty wells to the null effect was 0.076. Analysis of variance on the set of Hellinger distances for the empty controls to the null effect determined that the sum of squares for plate-level variation was 8.2 (140 degrees of freedom, p < 10^−15^), while the sum of squares for residual variation (i.e., biological or technical variation not due to plate-level variation) was 29.2 (6320 degrees of freedom). Thus, after non-control based non-parametric normalization, the majority of variation in absolute terms was not attributable to plate-to-plate variation. However, not all plate-to-plate variation could be eliminated.

### Hellinger Distance embedding vs KS statistic to null effect

When used to produce a univariate output (i.e., distance from each well fluorescence distribution to the null effect fluorescence distribution), both the Hellinger and KS metric distances were highly correlated (Pearson’s R = 0.96) (Additional file 8: Figure S6). The PC scores from the Euclidean embedding of Hellinger distances were compared with the values of the KS statistic from each well relative to the null effect, with the KS statistic treated strictly as a metric (distance) or allowed to be negative (so-called “signed KS statistic” in which a cumulative distribution function (CDF) to the right of the reference CDF is positive and to the left of a reference CDF is negative) (Additional file 9: Figure S7). The KS distance was highly correlated with the PC1 scores (R = 0.92), while the signed KS statistic was highly correlated with the PC2 scores (R = 0.97). However, neither the KS distance nor the signed KS statistic correlated strongly with the PC3 scores (R = 0.19 and 0.04, respectively) or any other higher order PC scores (data not shown).

### Control effect categorization

On each 384-well screening plate, there were a set of biological and technical control siRNAs chosen because they decreased (siGFP or siSox2 (gene id 20674)) [48] or appeared to increase (siNlk (gene id 18099)) GFP fluorescence based on preliminary studies (data not shown). siGFP and siSox2 dramatically reduced fluorescence (median Z-score of Hellinger distance to null distribution 5.51 and 4.61, respectively), while siNlk increased fluorescence but to a lesser degree (median Z-score 1.72).

**Fig. 4:**
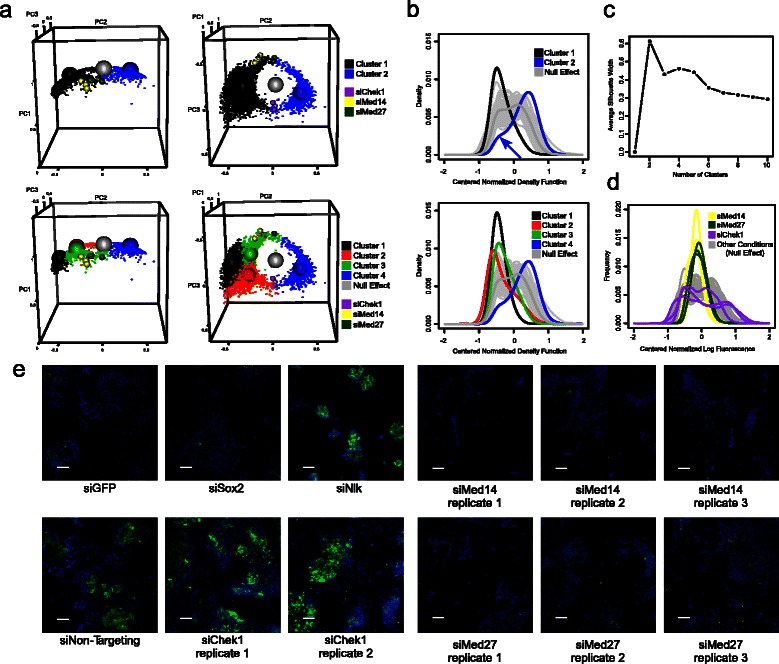
Outlier clustering to identify effect categories. **a** Plot of the first three PC weightings of all outlier conditions. The weightings are clustered around either 2 (top panels) or 4 (bottom panels) medoids, as suggested by the silhouette width criterion. Two views are shown on left and right. Distinct clusters are displayed in separate colors. Cluster centers are displayed as large spheres. Grey sphere is mean effect across all distributions. Non-outlier conditions are not displayed. The PC weightings of siMed14, siMed27 and siChek1 wells are displayed as small yellow, dark green and purple spheres, respectively. **b** Probability distribution functions corresponding to scaled centers from either 2- or 4-medoid clustering (top and bottom panels, respectively). The null effect probability distribution and a sample of mostly null effect distributions are shown for comparison (thick and thin grey lines, respectively). The medoid distributions are plotted in the same colors as panel a. A low-fluorescing subpopulation of cells in the probability distribution of cluster 2 conditions is noted with a blue arrow in the top panel. **c** Average silhouette width criterion, a measure of clustering appropriateness, following partitioning around medoids of first four PC weightings of outliers. **d** Probability distribution function of three technical replicates of siMed14, siMed27 and siChek1, shown in yellow, dark green and purple, respectively. A sample of 85 mostly null effect distributions are shown in grey. **e** Representative images for controls and outliers. Hoechst-stained nuclei are stained in blue and cytoplasmic GFP is in green. Controls: Conditions on every plate are chosen to decrease GFP (siSox2 and siGFP), increase GFP (siNlk) or have no effect (siNon-Targeting). Outliers: Technical replicates of siMed14, siMed27 and siChek1, which produce a narrower, narrower and wider GFP distribution, respectively. Scale bars (white) represent 80 μm

The majority of conditions from siGFP and siSox2 were significant outliers (|Z-score| > 2) when scored by conventional median cell fluorescence (769 of 809 siGFP, 743 of 769 siSox2 conditions and 287 of 633 siNlk conditions). A similar number of conditions were outliers when scored by our distribution methodology (787 of 809 siGFP, 699 of 769 siSox2 conditions and 264 of 633 siNlk conditions), confirming the screen’s consistency and our outlier identification ability (Fig. 3a, left panel). Representative images are shown in Fig. 4e.

The loss of pluripotency gene expression in siSox2 (rather than just GFP reporter expression in siGFP) drives cells to differentiate and disrupts cell-cell pluripotency signaling [48]. Careful visual examination of the images from siSox2 and siGFP (Fig. 4e) and their respective distributions (Fig. 3a, left panel and 3b) highlighted subtle distinguishing features consistent with this biology. We observed a more flattened cellular morphology in siSox2-treated wells compared with siGFP or siNon-target wells. In contrast, we observed small clusters of cells that continued to express GFP in siGFP conditions, consistent with their undisrupted cell-cell signaling. This difference is also reflected in the distributions of siSox2 vs siGFP (Fig. 3b).

### Clustering by distribution versus conventional scoring approaches

We assessed the feasibility of distinguishing the three distinct siRNA conditions using either our distribution-based methodology or one of several conventional scoring approaches. These conventional approaches included computation of Z-scores from the fluorescence of the median cell per well; computation of the KS statistic of each well to the null effect; and computation of an embedding from a parametric Gaussian mixture model representation of each distribution. We hypothesized that our distribution-based methodology would perform better than conventional approaches because it would capture subtle features only notable between pairs of distributions, particularly when the average levels of fluorescence in conditions were comparable, as in the siGFP and siSox2 treated wells.

We first clustered the median cell fluorescence values for each well from the 3 control siRNA types into 3 groups by partitioning around medoids. The 3 siRNA categories were assigned to their true group with 78 % accuracy, with siNlk controls very reliably assigned into a single group (Fig. 3c and Additional file 7: Table S1). However, because both siGFP and siSox2 conditions decreased median cell fluorescence to similar levels (Fig. 3c, left panel and 3a, middle panel), the distinction in the clustering between siSox2 and siGFP conditions was poor. Twenty percent of siGFP and 38 % of siSox2 conditions were incorrectly clustered.

We next clustered the effects computed by our distribution-based methodology (the “distribution scores”) to determine whether it could reliably distinguish such changes. When the first 4 PC weights of the distribution scores were used to cluster the effects into 3 categories, the distinction between siSox2 and siGFP controls was substantially improved compared with the conventional analysis (Fig. 3a, compare clustering assignment in right panel to true assignment in left panel, and Fig. 3c, right panel). The miscategorization rate of the 3 conditions decreased by 41 % compared with conventional analysis, bringing assignment accuracy to 87 %. Only 13 % of siGFP and 22 % of siSox2 conditions were incorrectly clustered, with nearly all siNlk conditions correctly clustered.

A clustering of the signed KS statistic (i.e., the KS statistic in which negative values are permitted) from the same controls to the null effect distribution performed even worse than the median fluorescence clustering, with 69 % assignment accuracy (Additional file 10: Figure S8).

To address the possibility that a parametric description of the distribution may be superior at distinguishing controls than the more general distributional approach, we fitted each siSox2, siGFP and siNlk control distribution to a 2-component Gaussian mixture model with unequal variances. A Euclidean embedding was constructed from the fitted parametric distributions using the Hellinger distance metric. When conditions were clustered into 3 categories as above (Additional file 11: Figure S9), the controls were distinguished with less accuracy than non-parametric distribution-based clustering. Moreover, each parametric distribution required the computationally intensive fitting of a non-linear model and 5, rather than 4, parameters.

Thus, while the median fluorescence levels associated with siGFP and siSox2 remain nearly the same (Fig. 3c, left panel), the distribution-based methodology reliably distinguishes between their effects and identifies the true experimental differences better than the most commonly applied conventional analyses.

### Hellinger Distance metric versus KS distance metric

**Fig. 5:**
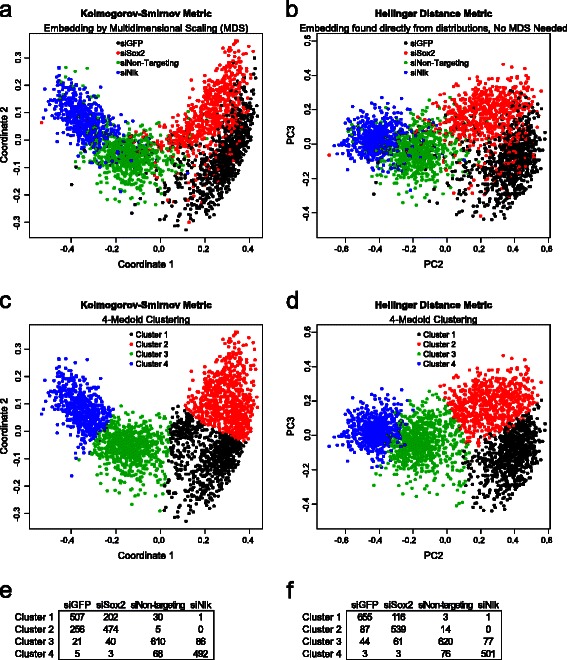
Comparison of Euclidean embedding constructed from either Kolmogorov-Smirnov distances or Hellinger distances. **a** For a subset of 2800 control wells, the Kolmogorov-Smirnov distance was computed for all pairs of distributions and multidimensional scaling (MDS) applied to compute a Euclidean embedding. The first two dimensions of the embedding were plotted for the given controls. **b** For the same controls, the Hellinger distances were used to directly generate a Euclidean embedding and the second and third PCs plotted (the first PC represents overlap with the null effect). The first 4 dimensions of the (**c**) Kolmogorov-Smirnov distance embedding and (**d**) the Hellinger distance embedding into 4 medoids were clustered and plotted as in panels a and b. Table of overlap between clustering and true identities of controls when using (**e**) Kolmogorov-Smirnov embedding or (**f**) Hellinger distance embedding

To specifically assess the effect of choice of distance metric within our methodology on the accuracy of clustering, we analyzed a more computationally tractable subset of the screen containing 2800 control wells representing the siGFP, siSox2, siNlk and siNon-targeting controls. We compared the Euclidean embedding derived from the Hellinger distances (found directly from the distributions) with the Euclidean embedding of KS distances (computed by MDS). When clustered using partitioning around 4 medoids (representing the 4 different control types), the first 4 dimensions of the embedding using the Hellinger distance clustered controls more accurately (83 %) than the first 4 dimensions of the embedding using the Kolmogorov-Smirnov distance (74 %) (Fig. 5). Therefore, in addition to its computational advantages, the Hellinger distance for embedding of distributions is also a better choice than the KS distance when using partitioning around medoids in this screen.

### Confidence threshold

We next identified a confidence threshold for the screen, i.e., the minimum biological variation that can be detected by the assay. We computed the Hellinger distances between all pairs of empty well control distributions. The median Hellinger distance between pairs of distributions from empty wells (i.e., conditions assumed to be identical) was 0.17, with 95 % of pairs of empty wells separated by <0.36 and 99 % of empty well pairs separated by <0.45. The median distance in Hellinger distribution space between members of the siSox2 cluster and members of the siGFP cluster (Fig. 3a, left panel) was substantially greater (0.36). Therefore, the difference between siSox2 and siGFP when measured by the Hellinger distance can be considered to be outside of the screen null-effect experimental confidence threshold.

In contrast, the median distance between the median cell log fluorescence for empty wells in the screen was 0.10, with 95 % of empty well pair distances <0.31. The median distance in median log fluorescence between members of the siSox2 cluster and members of the siGFP cluster (Fig. 3c, left panel) was 0.11, making the difference between siGFP and siSox2 essentially indistinguishable from experimental noise.

### Outlier identification, false positive rate and false negative rate

2384 of the 50339 non-control condition distributions, corresponding to 604 genes with consistent effects, were significantly distant from the null-effect distribution when the |Z-score| > 2 threshold was applied (Fig. 2c and d and Additional file 7: Tables S1 and Additional file 12: Table S2). Depletion of multiple known biological regulators of the pluripotent state, including *Sall4* and *T* (gene ids 99377 and 20997, respectively), differed significantly from the null distribution (Z-scores of 2.92 and 2.60, respectively). siRNA to retinoic acid receptors, expected to inhibit RA-mediated differentiation, effectively blocked the loss of fluorescence (Z-scores of 3.83, 1.94 and 2.00 for *Rxra* (gene id 20181), *Rxrb* (gene id 20182) and *Rxrg* (gene id 20183), respectively). Note that computation of Z-scores on distances to the null-effect distribution produces positive Z-scores for all large outliers regardless of whether the condition increases or decreases GFP.

We calculated the Z-score of Hellinger distance between the null-effect distribution and the distribution of each well. We then computed the median of this Z-score over all technical triplicate wells. 44 of 2154 triplicates of empty control wells had a median |Z-score| > 2 and 6 of 211 triplicates of siNon-targeting wells had a median |Z-score| > 2, yielding a false-positive rate of 2.0 % and 2.8 %, respectively. 5 of 216 triplicate siSox2 control wells had a median |Z-score| < 2 and 0 of 233 triplicate siGFP control wells had a median |Z-score| < 2, yielding a false-negative rate of 2.3 % and 0 %, respectively. 3.7 % of all tested non-control siRNA gene triplicates had a median |Z-score| > 2, suggesting (assuming a 2 % false positive rate) that 1.7/3.7 = 46 % of conditions identified as outliers are true positives when applying a |Z-score| > 2 cutoff.

### Screen outlier effect categorization

We next applied the same clustering strategy used for the controls to the outliers from the entire screen dataset, partitioning around medoids in their 4-dimensional best fit basis (Fig. 4a). Using the average silhouette width criterion [49], we found that 2 clusters were optimal, while 4 clusters were optimal if selection of 2 clusters was not permitted (Fig. 4c). There was also a clear decrease in the average silhouette width criterion with more than 5 peaks, suggesting the existence of between 3 and 5 meaningful but less clearly distinguishable probability distribution function categories. When we partitioned all outlier conditions using both 2 and 4 clusters, we observed a set of distinct effects among the clusters (Fig. 4b, top and bottom panels, respectively, and Additional file 7: Tables S1 and Additional file 12: Table S2).

The 2- and 4-cluster centers were converted back into probability distribution space (Fig. 4b), with each cluster center in Fig. 4a corresponding to an associated prototypical fluorescence distribution. The 2-center clustering divided outliers predominantly along their value in the second PC (Fig. 4a, right), representing, to a first approximation, increasing or decreasing fluorescence. Interestingly, the effects for conditions “typical” of these two cluster centers were not only confined to increasing or decreasing fluorescence. Conditions “typical” of cluster 1 (predominantly decreasing fluorescence) retained very few high-fluorescing cells. In contrast, conditions “typical” of cluster 2 (predominantly increasing fluorescence) contained a population of low-fluorescing cells (Fig. 4b, top, blue arrow). Similarly, the 4-medoid clustering identified effect categories roughly corresponding to low (cluster 1), bimodal low (cluster 2), medium-low (cluster 3) and bimodal high (cluster 4) fluorescence distributions (Fig. 4b, bottom). In short, clustering of outlier distributions identifies prototypical effects whose features are not adequately described in terms of increasing or decreasing fluorescence.

In order to extract functional information on the behavior of the genes in each cluster, we performed Gene Set Enrichment Analysis (GSEA) [50]. We identified a unique signature of gene ontologies specifically and significantly enriched (nominal p-value <0.05) in each cluster. For the 4-medoid clustering, we found, among others, that cluster 1 was significantly enriched for meiotic cell cycle genes; cluster 2 for genes associated with condensed chromosomes; cluster 3 for translation regulator activity, histone deacetylase complex and DNA helicase activity; and cluster 4 for negative regulation of cell cycle, negative regulation of cellular protein metabolic process and RNA pol II transcription factor activity ontologies (Additional file 13: Figure S10 and Additional file 14: Table S3). Thus, gene ontology analysis can be applied to distribution-based effect clusters to reveal potentially unappreciated functional connections between sets of genes that produce similar effects.

### Validation of selected high-confidence outliers

We sought to demonstrate the advantages of our methodology by confirming the biological role of a selected subset of high-confidence outlier genes (HCOs) that were not identified by the conventional analysis. To limit false positives (as described above), we required that HCO conditions be represented as outliers, i.e., |Z-score| > 2 of Hellinger distance to the null effect in 4-dimensional basis, in at least 2 out of 3 technical replicates. To exclude outliers with inconsistent effects, we also required that the Hellinger distance between at least one pair of outlier distributions for a particular gene was smaller than the distance of either distribution to the null effect. We applied the above HCO criteria to our *Nanog* reporter ESC siRNA screen and identified conditions affecting the distribution of GFP fluorescence (Additional file 7: Tables S1 and Additional file 12: Table S2).

Among the HCO genes identified based on distributional changes, we observed GFP-reducing effects of siBrd4 (gene id 57261) (median distribution Z-score 4.58, median fluorescence Z-score −2.49) and siSnai1 (gene id 20613) (median distribution Z-score 3.07, median fluorescence Z-score 2.10) as well as a GFP-increasing effect of siSnai2 (gene id 20583) (median distribution Z-score 2.34, median fluorescence Z-score 2.40). The antagonistic effects of Snai1 and Snai2 in control of pluripotency have been recently reported [12] as has a confirmation of the role of Brd4 in ESCs [51–53]. While these particular hits were identified previously only using a median fluorescence-based analysis [12], our distribution-based approach captured them equally well.

We also identified a number of HCO genes that would have been missed with a conventional effects ranking based on median cell fluorescence. Among them we found that siRNA pools targeting *Chek1* (gene id 12649) mRNA had a minimal effect on the median GFP fluorescence (median Z-score 0.47) but significantly affected the fluorescence distribution (median Z-score > 2), leading to a broader distribution including more higher-fluorescing cells (Figs. 4d and 4e). The signed KS statistic of siChek1 to the null effect was also not significant (median Z-score −1.14).

**Fig. 6:**
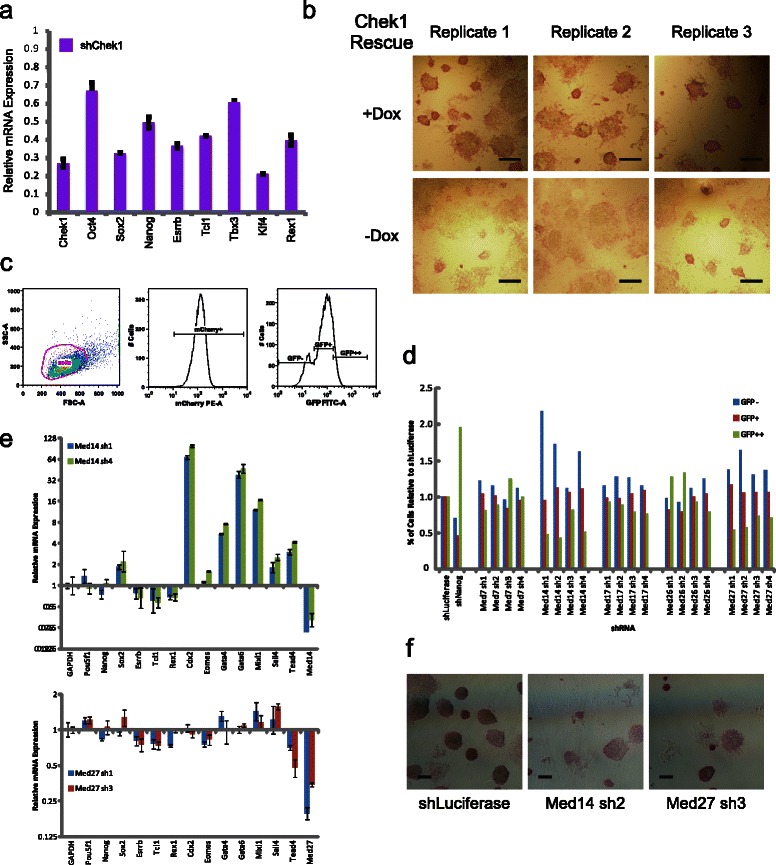
Effects of Chek1 and Mediator mRNA depletion on pluripotency. **a** mRNA changes following shRNA depletion of Chek1 relative to shLuciferase levels. Error bars are mean ± s.d.. **b** Alkaline phosphatase staining of a Chek1 rescue line in which exogenous doxycycline-inducible expression of Chek1 rescues endogenous depletion. Cells are stained for AP activity following 5 days with (+) and without (−) doxycycline (Dox). Scale bars (in black) represent 200 μm. **c** Flow cytometry analysis framework, as applied to shLuciferase-transduced NG4 cells. GFP expression of puromycin-resistant mCherry + cells is classified into GFP-, GFP+ and GFP++ categories, representing GFP-negative, GFP-low and GFP-high cell populations, respectively. **d** Effects of Mediator gene shRNA depletion on NG4 reporter fluorescence distribution. **e** mRNA changes following shRNA depletion of Med14 and Med27 relative to shLuciferase levels. Error bars are mean ± s.d. **f** Alkaline phosphatase staining following shRNA depletion of Mediator genes. Scale bars (in black) represent 200 μm

To address the potential role of *Chek1* in pluripotency, we constructed short hairpin (sh) RNA lentiviruses targeting endogenous *Chek1* mRNA and measured changes in pluripotency gene mRNA levels. We found that after depleting Chek1 levels by 73 %, the expression of numerous pluripotency genes, including *Sox2* and *Nanog*, was depleted by more than 50 % relative to a shLuciferase control (Fig. 6a).

To confirm these effects, we utilized a previously described genetic complementation ”rescue” system [5, 23]. Here, endogenous *Chek1* mRNA is constitutively depleted by shRNA and gene expression rescued by exogenous shRNA-insensitive doxycycline (Dox)-inducible Chek1. We performed alkaline phosphatase (AP) staining 5 days after removal of Dox. Chek1 depletion in the -Dox condition dramatically reduced the number of AP-positive colonies relative to the + Dox Chek1 maintenance condition, indicative of compromised pluripotency (Fig. 6b). These connections are supported by previous reports on *Chek1* [54].

We also noted the presence of multiple Mediator gene members in the HCO list, including *Med7*, *Med14*, *Med17*, *Med26* and *Med27* (Fig. 3a, gene ids 66213, 26896, 234959, 70625 and 68975, respectively). Of these genes, *Med14*, *Med17* and *Med27* would not have been noted as outliers when scored by the median cell fluorescence parameter. For example, effects of siRNA pools targeting *Med14* and *Med27* on the median cell fluorescence (conventional criteria) were non-significant in our screen (median Z-score of −1.33 and −1.02, respectively, across 3 technical replicates). In addition, in a *Pou5f1* pluripotency reporter screen that assayed multiple Mediator genes, only 1 of 5 shRNAs in the library targeting *Med27* scored as significant (Z-score < −2) by the average cell fluorescence criterion used [47]. Although siMed14 was significant by signed KS statistic to null effect (median Z-score 2.34), siMed27 was not (median Z-score 1.60). In contrast, the effect of siRNAs targeting *Med14* and *Med27* was clear in our *Nanog* reporter screen scored by the distribution-based methodology (median Z-score of 3.54 and 2.97, respectively). siMed14- and siMed27-treated cells were characterized by atypically uniform but intermediate reporter fluorescence (Figs. 3d, 3e and Additional file 7: Tables S1 and Additional file 12: Table S2).

Several studies have demonstrated that Mediator complex members regulate transcription of the pluripotency gene, *Pou5f1* [47, 55]. In addition, Med12 has been reported to bind to the *Nanog* promoter [47]. We therefore tested the hypothesis that loss of other Mediator components might regulate *Nanog* promoter activity. In order to assess the effects of several uncharacterized Mediator components on the ability of *Nanog*-promoter GFP cells to sustain Nanog expression, we constructed 4 shRNA lentiviruses targeting distinct regions of the corresponding mRNA and 3’UTR of each of the Mediator components *Med7*, *Med14*, *Med17*, *Med26* and *Med27* and transduced NG4 mESCs with them along with shLuciferase and shNanog controls. As expected in light of Nanog’s negative regulation of its own promoter [56], repressing Nanog led to an increase in *Nanog* promoter-driven fluorescence (Fig. 6c and d). Surprisingly, we found that shRNAs targeting *Med14* and *Med27* led to a dramatic increase in the fraction of GFP-negative cells relative to Luciferase shRNA. Similar but less dramatic effects were noted for *Med17* shRNAs. In all of these cases, Mediator gene knockdown was accompanied by a decrease in the high-expressing GFP fraction (noted as GFP++).

For *Med14* and *Med27*, we confirmed the knockdown of target genes by reduction in mRNA levels (Fig. 6e). We observed that depletion of both *Med14* and *Med27* mRNA led to a significant reduction in mRNA of the pluripotency genes *Esrrb*, *Tcl1* and *Rex1* (gene ids 26380, 21432 and 22702, respectively)*.* Further, ESCs treated with *Med14* shRNA strongly up-regulated the lineage markers *Cdx2*, *Gata4*, *Gata6*, *Mixl1*, *Sall4* and *Tead4* (Fig. 6e, gene ids 12591, 14463, 14465, 27217, 99377 and 21679, respectively). Alkaline phosphatase staining on the shRNA-treated cells demonstrated a marked loss of activity relative to the shLuciferase control (Fig. 6f).

We thus find that conditions affecting the *Nanog*-promoter driven fluorescence single-cell distribution can be reliably identified from high-content screening data. Several biologically relevant regulators of the pluripotent state were missed by conventional analysis approaches but identified by our distribution-based methodology.

## Discussion

The principal utility of our distribution-based methodology stems from its ability to capture all types of changes to a population of reporter cells. While the conventional mean or median cell parameter approach to identifying outliers in a screen is sufficient to identify conditions that dramatically shift all cells to higher or lower values, it would not capture effects that, for example, lead to a high-variance distribution with more cell parameter levels at the extremes or a highly uniform intermediate one.

We find that even scoring effects using the univariate KS statistic to the null effect (Additional file 10: Figure S8) is inadequate. The KS statistic most notably failed to identify two of the three hits from our methodology (siChek1 and siMed27) that we experimentally proved. The lack of correlation of the widely used KS statistic with PC3 Z-scores (Additional file 9: Figure S7) is one of its key shortcomings, particularly when conditions that primarily affect PC3 scores are of biological interest.

Analyses of effects in a space determined by metric distance between probability distribution functions address the above deficiencies. Conditions with strong distributional effects are clearly captured as outliers based on the Hellinger distance metric, whether or not they produce a significant “up or down” effect on the median or mean fluorescence levels. While our results suggest the superiority of the Hellinger distance relative to the KS distance when used in partitioning around medoids of the Euclidean embedding (Fig. 5), we do not exclude the possibility of an alternative clustering methodology (possibly in conjunction with an alternative distance metric) permitting even better clustering of screen effects. However, given its obvious computational advantages and strong performance in clustering, we consider selection of the Hellinger distance to be adequate for most practical applications.

Our methodology can be extended to multivariate data. For example, the Euclidean embedding for the parameter A distribution *(r*_*1*_ *, r*_*2*_ *,…,r*_*m*_ *)* can be joined with the embedding for the parameter B distribution *(r’*_*1*_ *, r’*_*2*_ *,…,r’*_*m*_ *)* measured from the same cells in the same well as a higher-dimensional embedding with weighting *(ar*_*1*_ *, ar*_*2*_ *,…,ar*_*m*_ *, br’*_*1*_ *, br’*_*2*_ *,…,br’*_*m*_ *)* to be treated with dimensionality reduction and clustering as above. Although assumptions of equal variance (i.e., a = b) are widely made [26], alternative and possibly superior relative weights exist.

It is often of interest to understand the response of a gene-product (such as Nanog) across conditions in order to appreciate its biological function. The median or mean parameter value cannot adequately describe how individual cells respond to a given perturbation. A parametric description (such as a Gaussian mixture model) makes potentially unwarranted assumptions about the possible distributions. The lack of superior performance of a Gaussian mixture model may be expected in light of the limitations of parametric models, including over-fitting, suboptimal fitting and sensitivity of clustering to assumptions of normality [57]. In contrast, our method can automatically determine all significant distribution-level responses of a reporter and classify them into a desired number of clusters, taking advantage of the library diversity to better understand the reporter.

The selection of optimal cluster number (in our case, 2 vs 4 vs something else) remains a matter for debate. Two clusters is the optimal number based on the silhouette width criterion in our dataset (Fig. 4c) and divides the data into its most “natural” grouping, to first order splitting effects into those that increase or those that decrease GFP. However, if the intention of this analysis is to find sets of conditions that affect GFP in slightly different yet detectable ways, then greater than 2 clusters is required. The number of clusters greater than 2 that maximizes the silhouette width criterion in this study is 4, although using 3–5 clusters is nearly as good (Fig. 4c). This methodology may be applied to both aims: demonstrating that the effects can be broadly grouped into a given number of optimal categories (e.g., 2); or to cluster effects into a less optimal but larger number of categories suggested by the next-best silhouette width criterion (e.g., 4) or a biological mechanism.

Our pluripotency siRNA screen analysis also demonstrates the successful application of our distribution-based approach to its primary aim, namely quantifying all effects and isolating the most interesting ones. Biologically distinct conditions that appear to have similar levels of median fluorescence can be distinguished with far greater accuracy when each condition is viewed as affecting the distribution (Fig. 3). The discrimination between siSox2 and siGFP clearly demonstrates that differences in parameter distributions can identify meaningful differences that are not adequately captured by the parameter median values.

## Conclusions

High-content screening offers single-cell data for each condition. In order to derive greater biological meaning from high-content screens, a screening methodology must effectively identify biologically interesting conditions and distinguish them from one another. Comparing conditions by the mean or median reporter value (e.g., fluorescence) or by using any methodology that provides only a univariate output needlessly discards biologically relevant information. Our methodology treats cells in each condition as being sampled from an underlying distribution. We distinguished the effects of known controls from each other better than alternative approaches. We also identified several gene-products with confirmed biological effects that would have been missed by other approaches. We therefore foresee routine application of our methodology in analysis of future high-content screens.

## Methods

### RNAi library and plate preparation

Our screen was conducted as previously described [12].

### Cell-level data extraction

Confocal images taken in blue (Hoechst 33342 nuclear staining) and green (GFP) channels were processed for cell segmentation analysis with the MetaXpress software package as previously described [12, 58]. Nuclei were segmented using the blue channel with a minimum width of 8 μm (10 pixels) and a maximum width of 26 μm (33 pixels). Cytoplasmic regions were segmented using the GFP channel with a minimum width of 10 μm (13 pixels) and a maximum width of 30 μm (38 pixels). For each image, we extracted average fluorescence per cell over the entire cytoplasmic area (Fig. 1c).

Average fluorescence values per cell for each condition were exported as text files, which were grouped into separate files for each well with Perl.

### Software used in methodology

All further normalization, density estimation, matrix operations and plotting were performed in R [59]. All scripts and code used in this paper but not included in DistributionAnalyzer are available from the authors upon request.

### Cell culture

For the RNAi screen, cell culture and imaging were performed as described previously [12]. Additional cell culture was performed as described previously [5, 23, 54, 56]. NG4, CCE and Ainv15 mESC lines were used as previously described [11]. Cells were maintained feeder-free on gelatin-coated tissue culture dishes in 15 % fetal bovine serum ESC culture media. Ainv15 rtTA expressing ESCs used to generate rescue clones were maintained on primary mouse embryonic fibroblasts (MEFs) in ESC culture media supplemented with doxycycline (2 μg/ml).

### Microscopy

Images were acquired using an ImageXpress Ultra high-content confocal microscope (Molecular Devices). For each well, 4 non-overlapping images were collected with 2x binning with 20x objective as 1000x1000 pixel 16-bit files. Each image corresponded to an 800 μm x 800 μm area.

For the representative (1000x1000 pixel) images shown in Fig. 4e, background levels from a 200x200 pixel blur were subtracted from the composite blue and green images and image intensities scaled by 4 using ImageMagick. To remove spurious background pixels, a 5x5 pixel blur mask was constructed and thresholded at 3 % of the image range. All pixels in the image below the threshold were set to black.

The AP-stained images for Chek1 rescue clones were taken with a Nikon TE2000-U and 4x objective. Images were then adjusted in GIMP for color balance (+100 towards blue) and saturation (−40).

### shRNA design, lentivirus generation, and mouse ESC transduction

shRNAs utilized in this study are listed in Additional file 15: Table S4. Oligonucleotides encoding each shRNA duplex were synthesized by Integrated DNA Technologies and cloned into the AgeI/EcoRI sites of the lentiviral-based shRNA expression vector pLKO.pig (pLKO.1 PuroR-IRES-GFP) or pLKO.pim (pLKO.1 PuroR-IRES-mCherry) following the supplier’s protocol (Addgene) [60]. All shRNA constructs were confirmed by sequencing. The pLKO.pig Chek1 shRNA1 was used for generating a rescue clone, as described previously [5, 23].

Lentiviruses were generated in HEK-293 T cells by Superfect-mediated cotransfection of lentiviral-based shRNA plasmids and the pCMV-dR8.2 (packaging) and pCMV-VSVG (envelope) plasmids. Viral supernatants were concentrated using Amicon Ultra centrifugal filter units (Millipore) at 1600 g for 20 min. and stored at −80 °C. For infection, mESCs were infected in media supplemented with polybrene (8 μg/mL; Sigma). Cells were incubated overnight with virus and subsequently cultured in fresh media for 4 days. Infected cells were cultured in media supplemented with 2 μg/mL puromycin for an additional 4 days, after which mRNA was extracted.

### Alkaline phosphatase (AP) staining

AP staining was measured using an Alkaline Phosphatase Staining Kit (Stemgent) following the manufacturer’s recommendations. For Mediator shRNAs, AP Staining was performed 7 days after puromycin selection.

### Quantitative RT-PCR analysis

RNA was extracted using Trizol and the RNeasy Mini Kit (Qiagen). 1 μg of total RNA was converted into double-stranded cDNA using the High Capacity reverse transcription kit (Applied Biosystems). Quantitative PCR was performed using the Fast SYBR® Green Master Mix (Applied Biosystems) on the LightCycler480 Real-Time PCR System (Roche). Gene-specific primers used for this study were described previously [54].

### Availability of supporting data

The datasets supporting the results of this article are included within the article and its supplementary files.

## Additional files

Additional file 1: Code S1. R source code and sample data for distribution-based high-content screen analysis
Additional file 2: Figure S1. Effects of non-parametric data acquisition normalization and kernel density estimation. (**a**) Boxplot of the median cell fluorescence for each condition before and after  non-control based non-parametric transformation for 25 screened plates. All cells in a screening plate are assigned a fluorescence based on the fluorescence of the cell in the reference plate with the closest quantile. Box line denotes median condition, box ends denote first and third quartiles and whiskers are located at 1.5 times the interquartile range. The median cell fluorescence for conditions outside this range is plotted. (**b**) The median cell fluorescence for each condition (well) in two sets of screening plates before and after non-parametric transformation. Conditions with outlier median cell fluorescence remain as outliers after transformation. (**c**) Log fluorescence of 1000 cells derived from image-based cell segmentation from siRNA screen are normalized as above and displayed as a histogram (left) or converted to a probability distribution function using kernel density estimation (right).
Additional file 3: Figure S2. Alternative normalization procedures and effect on fluorescence distributions. The collection of cells on a screening plate is normalized by one of three procedures: (**a**) affine parametric transformation by computing the Z-score of the single cell log fluorescence (**b**) control-based non-parametric normalization of the cells from control wells in each plate to the control wells of the reference plate (Plate 1), with interpolation for all non-control cells; and (**c**) non-control based non-parametric normalization of the all the cells in each plate to all the cells of the reference plate (Plate 1), with interpolation. The fluorescence probability distributions of the same set of select controls (siGFP, siSox2 and empty) from Plate 1 (solid lines) and Plate 2 (dashed lines) are plotted together (**d**) before normalization, **(e)** after Z-score normalization, (**f**) after control-based non-parametric normalization and (**g**) after non-control based non-parametric normalization. 
Additional file 4: Figure S3. Plate-level and well-level variation of control wells. **(bottom)** The Hellinger distance to null-effect was computed for all empty control wells (expected to have no effect) (blue) and all siGFP wells (expected to deplete GFP and substantially alter the fluorescence distribution) (red). For the control wells of each plate, a boxplot was computed. Box line denotes median value, box ends denote first and third quartiles and whiskers are located at 1.5 times the interquartile range. Values outside this range are plotted as empty circles. **(top)** Student’s t -test was computed between the Hellinger distance scores of siGFP control wells and empty control wells for each plate. Negative log of the p-values of the *t*-test are plotted for each plate.
Additional file 5: Figure S4. Error in estimating the probability distribution as a function of number of cells per well. The Kolmogorov-Smirnov statistic was computed between the actual cell fluorescence values and the estimated probability distribution for each well in the screen. This statistic serves as a marker of error in the process of estimating the distribution. The statistic was plotted as a function of the number of cells in the well used to generate the distribution.
Additional file 6: Figure S5. Error in estimating the probability distribution as a function of number of bins used in estimation. For all wells in a screening plate, the number of equally spaced bins (*m*) at which the probability distribution was estimated was varied, with *m* = 256 (left), 512 (center) and 1024 (right). The error in the estimation of the probability distribution was measured for each well as the Kolmogorov-Smirnov statistic between the actual cell fluorescence values and the estimated probability distribution.
Additional file 7: Table S1. All effects in the genome-scale RNAi screen. All 62252 conditions (wells) with >100 wells are scored for median fluorescence per condition; Z-score Hellinger distance to the null effect; and in the 4-dimensional best-fit basis of the Hellinger-distance embedding, i.e., the distribution-based PC scores. For 2- and 4-medoid cluster assignment, 0 indicates non-outlier and cluster membership is noted from 1 to 2 or 1 to 4.
Additional file 8: Figure S6. Difference between Hellinger distance to null effect and Kolmogorov-Smirnov distance to null effect. The Hellinger distance of all treated wells in the screen to the null effect is plotted against the Kolmogorov-Smirnov distance between the same wells and the null effect. The distance metrics are very highly correlated.
Additional file 9: Figure S7. Comparing the Kolmogorov-Smirnov statistic to null effect and the first three PC scores of the Hellinger distance embedding. The Kolmogorov-Smirnov distance (always positive) (top) and the signed Kolmogorov-Smirnov statistic (possibly negative) (bottom) was computed for all wells in the screen and plotted against the first three PC scores of the Euclidean embedding of Hellinger distances. The third PC scores from the Hellinger distance embedding (right) are not substantially correlated with the Kolmogorov-Smirnov distances to null effect.
Additional file 10: Figure S8. Clustering controls using Kolmogorov Smirnov score to null effect. For each control well, the signed Kolmogorov-Smirnov distance was calculated between the cumulative distribution function of the cells in the well and the null-effect. Values are ordered by category (siNlk, siGFP and siSox2) and separated by vertical bars. Clustering with 3 medoids of the Kolmogorov-Smirnov scores (left) is compared to 3 medoid clustering by Hellinger distance (right).
Additional file 11: Figure S9. Clustering controls using dual Gaussian mixture model. Median cell fluorescence for all control siRNA conditions that could be fit to convergence with a dual Gaussian mixture model. Values are ordered by category (siNlk, siGFP and siSox2) and separated by vertical bars. Clustering with 3 medoids by Hellinger distance between distributions (center) or by Hellinger distance between distribution fit to a dual Gaussian mixture model (right) is shown in magenta, blue and orange. Number of category conditions assigned to each cluster is shown as numbers beneath the category.
Additional file 12: Table S2. Effects in the genome-scale RNAi screen grouped by unique condition. Conditions from Additional file 7: Table S1 were grouped into 16784 unique siRNA pools (each targeting a different gene) and the median applied over replicates. Replicates with no mode whose distributions fell into different clusters or the non-outlier cluster are assigned to the non-outlier cluster. The PC scores were averaged. 
Additional file 13: Figure S10. Selected gene ontologies enriched in 4-medoid clustering of distribution effects. Gene set enrichment analysis was performed on the unique mapped genes assigned to each outlier cluster distribution from a 4-medoid clustering. A selected subset of motif gene sets in the Molecular Signatures Database with a nominal p-value <0.05 is shown here. 
Additional file 14: Table S3. Gene sets enriched in the 4-medoid distribution clusters. Gene set enrichment analysis was performed on the unique mapped genes assigned to each outlier cluster distribution from a 4-medoid clustering. The tested motif gene sets in the Molecular Signatures Database are shown here. 
Additional file 15: Table S4. shRNA sequences used in this study.
