# Supplementary material for: Distribution Analyzer, a methodology for identifying and clustering outlier conditions from single-cell distributions, and its application to a Nanog reporter RNAi screen
Source: BMC Bioinformatics. 2015 Jul 22;16:225. doi: 10.1186/s12859-015-0636-7 (PMC4511455; doi:10.1186/s12859-015-0636-7)
Supplement: Additional file 2: Figure S1. — Effects of non-parametric data acquisition normalization and kernel density estimation. (a) Boxplot of the median cell fluorescence for each condition before and after non-control based non-parametric transformation for 25 screened plates. All cells in a screening plate are assigned a fluorescence based on the fluorescence of the cell in the reference plate with the closest quantile. Box line denotes median condition, box ends denote first and third quartiles and whiskers are located at 1.5 times the interquartile range. The median cell fluorescence for conditions outside this range is plotted. (b) The median cell fluorescence for each condition (well) in two sets of screening plates before and after non-parametric transformation. Conditions with outlier median cell fluorescence remain as outliers after transformation. (c) Log fluorescence of 1000 cells derived from image-based cell segmentation from siRNA screen are normalized as above and displayed as a histogram (left) or converted to a probability distribution function using kernel density estimation (right). [file 12859_2015_636_MOESM2_ESM.pdf]

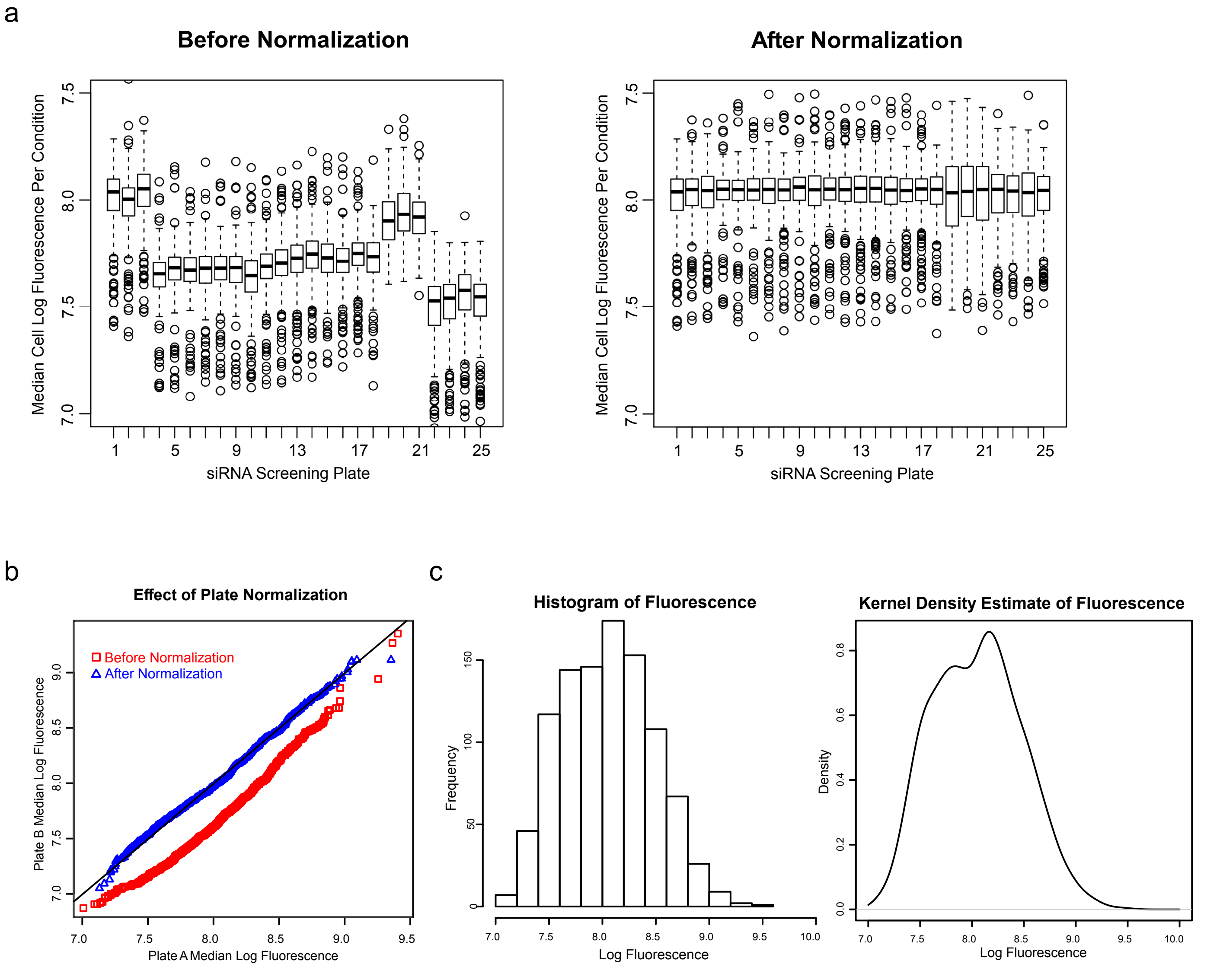
a
Before Normalization
After Normalization
Median Cell Log Fluorescence Per Condition
7.0
7.5
8.0
7.5
1
5
9
13
17
21
25
siRNA Screening Plate
b
Effect of Plate Normalization
Before Normalization
After Normalization
Plate B Median Log Fluorescence
7.0
7.5
8.0
8.5
9.0
9.5
Plate A Median Log Fluorescence
c
Histogram of Fluorescence
Frequency
0
50
100
150
Log Fluorescence
10.0
Kernel Density Estimate of Fluorescence
Density
0.0
0.2
0.4
0.6
0.8
Log Fluorescence
