# Supplementary figures and images for: Distribution Analyzer, a methodology for identifying and clustering outlier conditions from single-cell distributions, and its application to a Nanog reporter RNAi screen
Source: BMC Bioinformatics. 2015 Jul 22;16:225. doi: 10.1186/s12859-015-0636-7 (PMC4511455; doi:10.1186/s12859-015-0636-7)

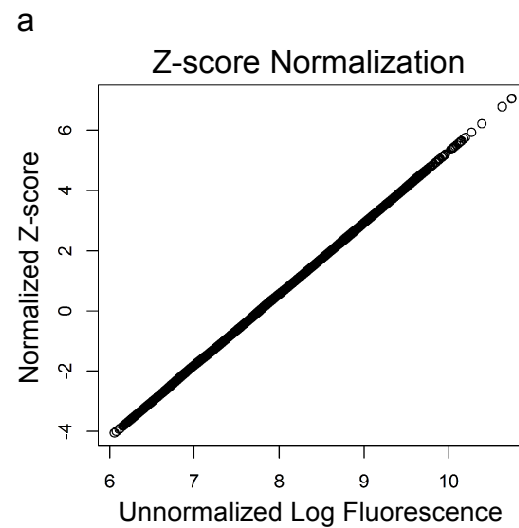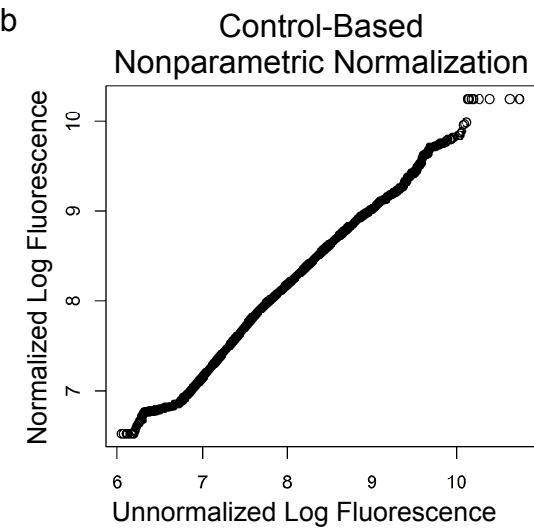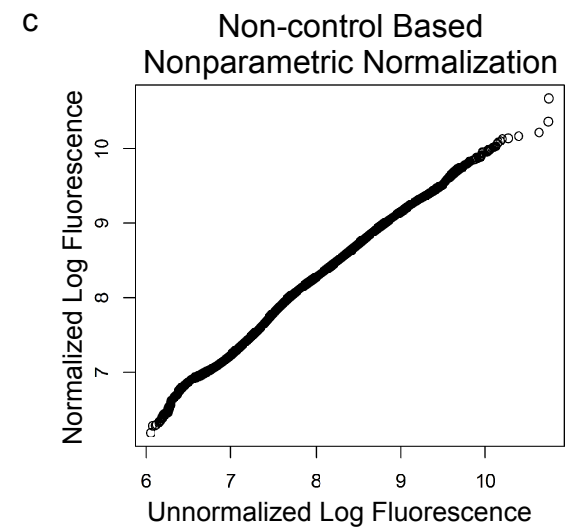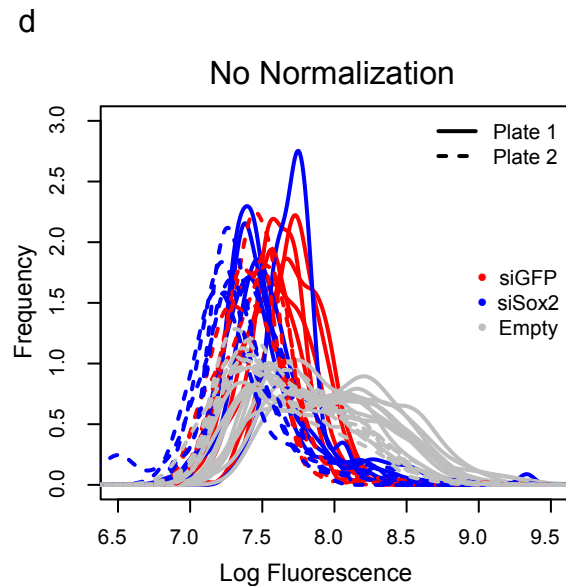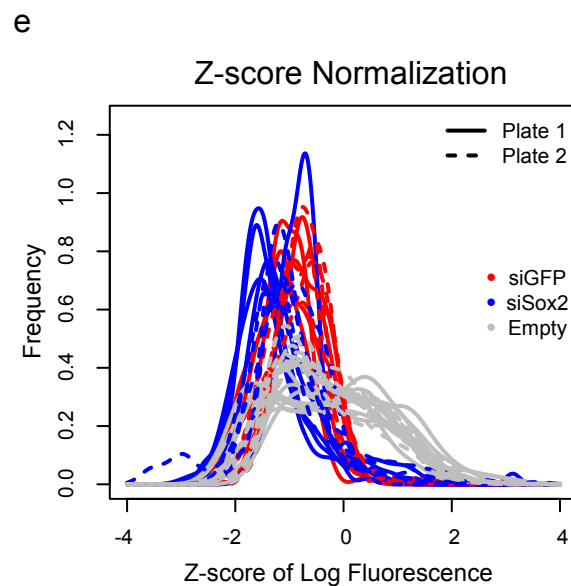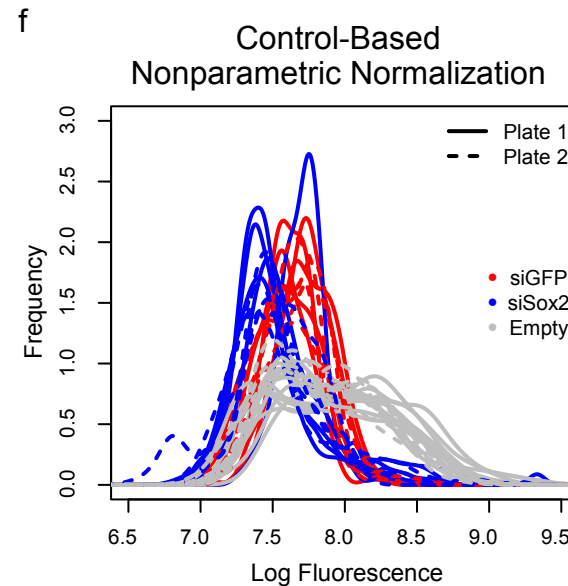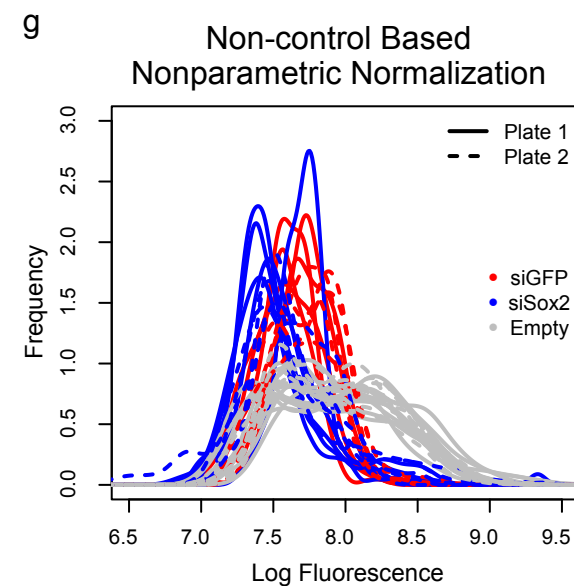

Supplement: Additional file 3: Figure S2. — Alternative normalization procedures and effect on fluorescence distributions. The collection of cells on a screening plate is normalized by one of three procedures: (a) affine parametric transformation by computing the Z-score of the single cell log fluorescence (b) control-based non-parametric normalization of the cells from control wells in each plate to the control wells of the reference plate (Plate 1), with interpolation for all non-control cells; and (c) non-control based non-parametric normalization of the all the cells in each plate to all the cells of the reference plate (Plate 1), with interpolation. The fluorescence probability distributions of the same set of select controls (siGFP, siSox2 and empty) from Plate 1 (solid lines) and Plate 2 (dashed lines) are plotted together (d) before normalization, (e) after Z-score normalization, (f) after control-based non-parametric normalization and (g) after non-control based non-parametric normalization. [file 12859_2015_636_MOESM3_ESM.pdf]

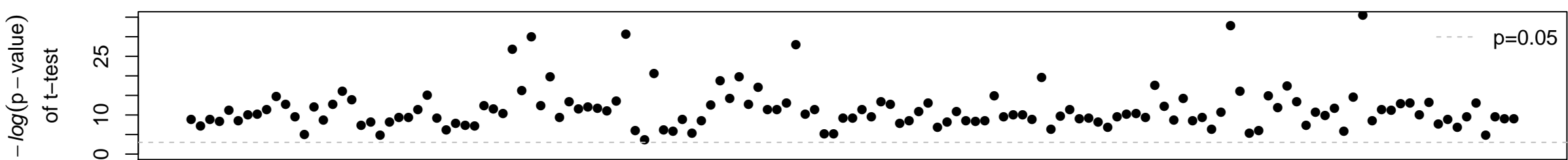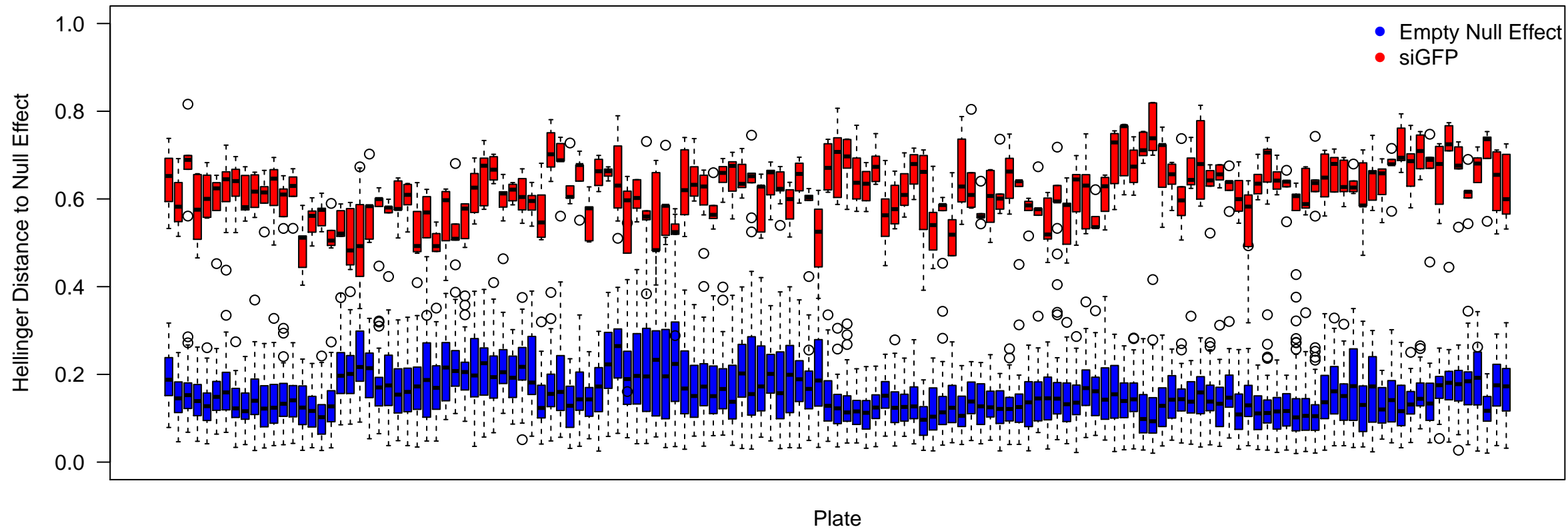

Supplement: Additional file 4: Figure S3. — Plate-level and well-level variation of control wells. (bottom) The Hellinger distance to null-effect was computed for all empty control wells (expected to have no effect) (blue) and all siGFP wells (expected to deplete GFP and substantially alter the fluorescence distribution) (red). For the control wells of each plate, a boxplot was computed. Box line denotes median value, box ends denote first and third quartiles and whiskers are located at 1.5 times the interquartile range. Values outside this range are plotted as empty circles. (top) Student’s t -test was computed between the Hellinger distance scores of siGFP control wells and empty control wells for each plate. Negative log of the p-values of the t-test are plotted for each plate. [file 12859_2015_636_MOESM4_ESM.pdf]

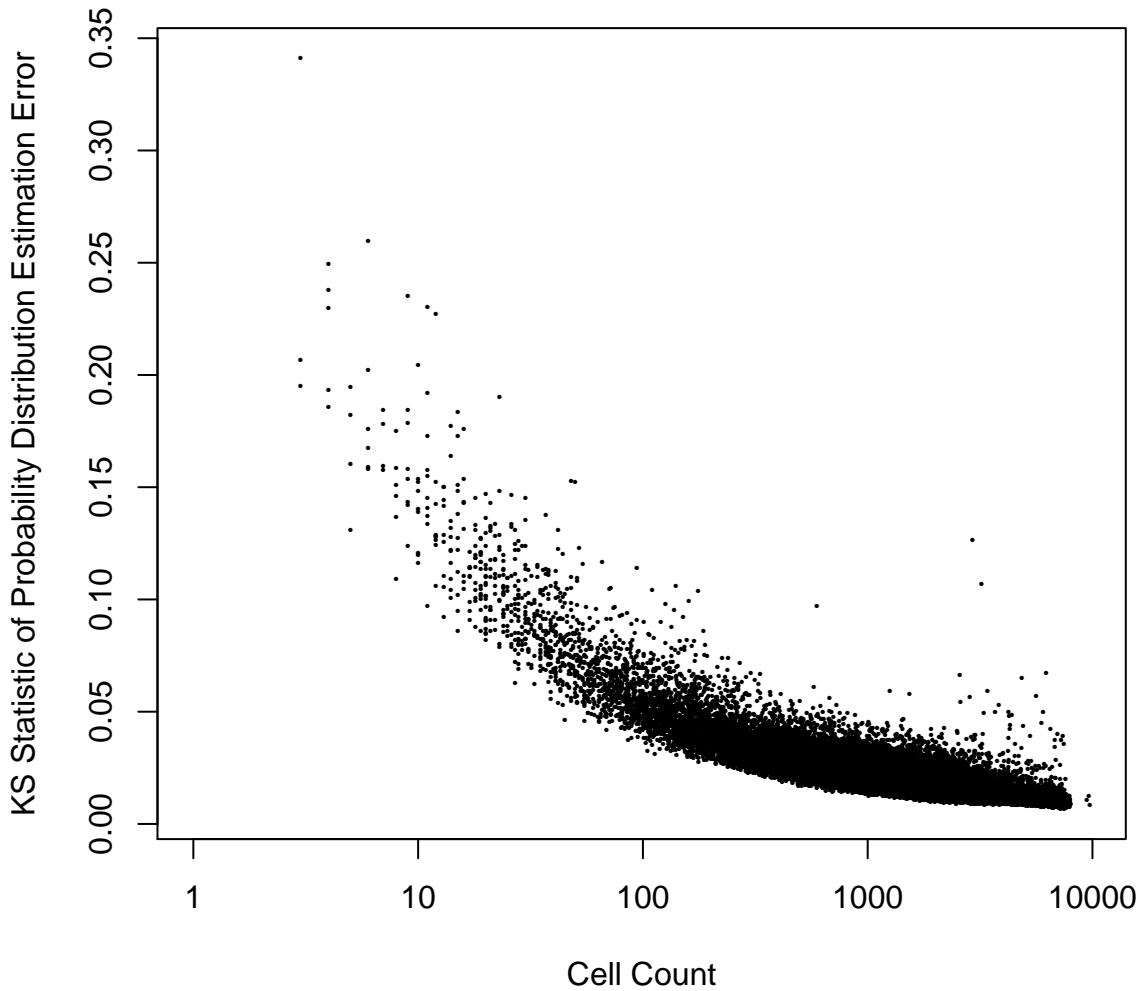

Supplement: Additional file 5: Figure S4. — Error in estimating the probability distribution as a function of number of cells per well. The Kolmogorov-Smirnov statistic was computed between the actual cell fluorescence values and the estimated probability distribution for each well in the screen. This statistic serves as a marker of error in the process of estimating the distribution. The statistic was plotted as a function of the number of cells in the well used to generate the distribution. [file 12859_2015_636_MOESM5_ESM.pdf]

# Error in Estimation of Probability Density Function

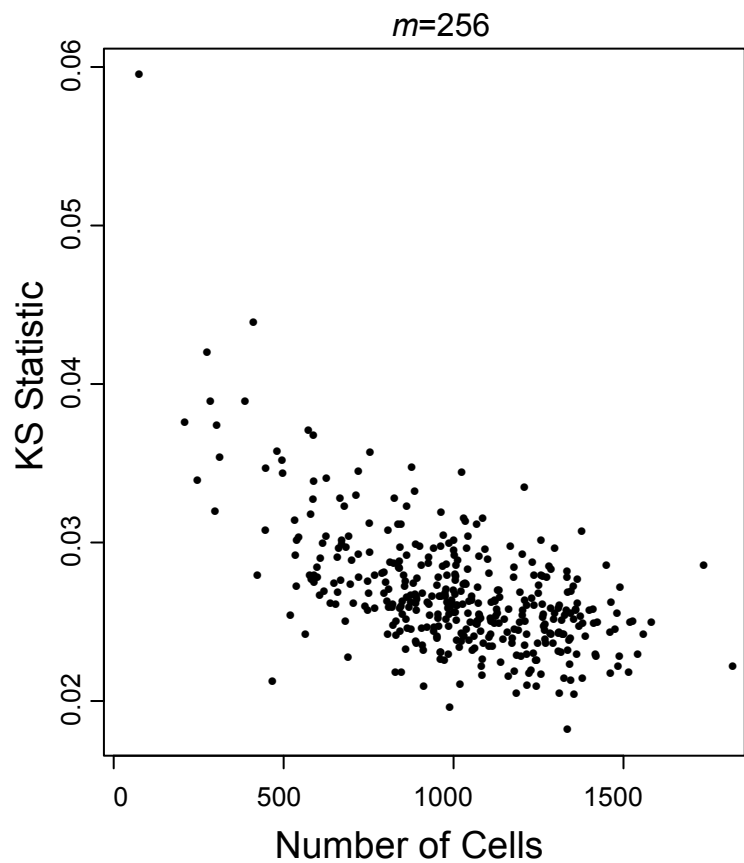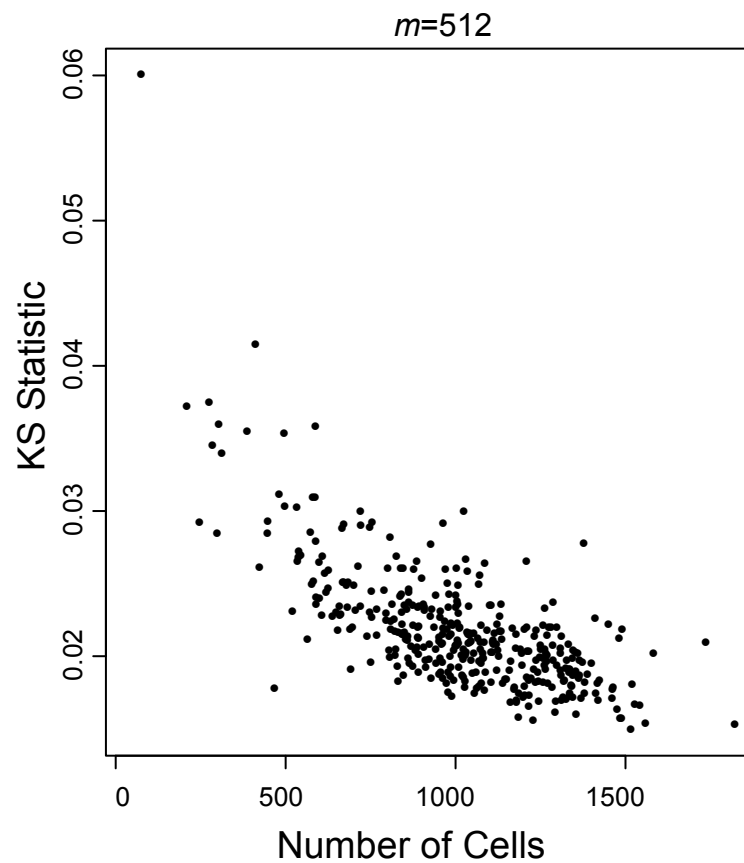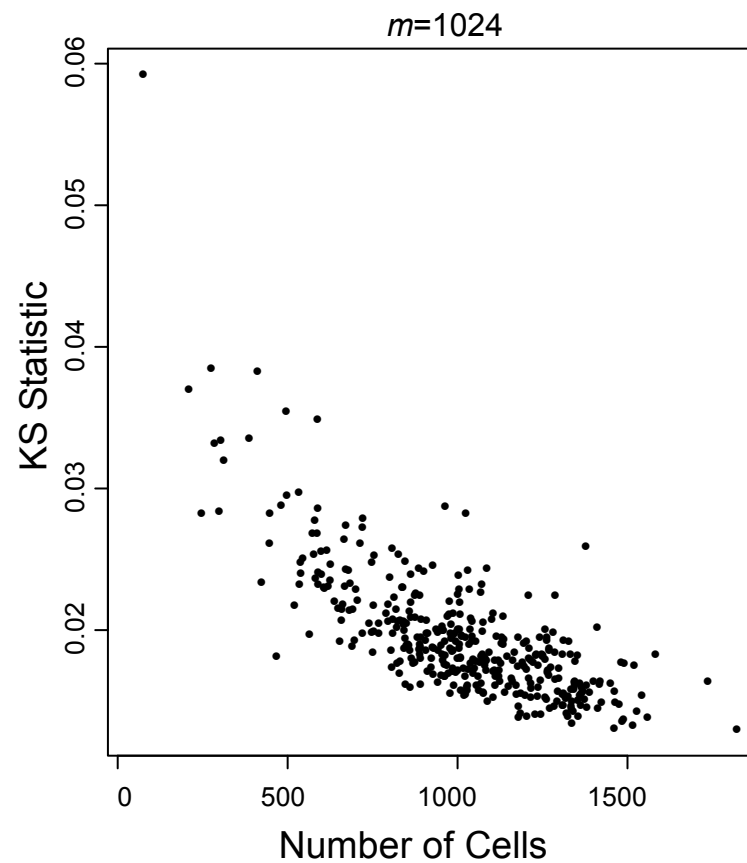

Supplement: Additional file 6: Figure S5. — Error in estimating the probability distribution as a function of number of bins used in estimation. For all wells in a screening plate, the number of equally spaced bins (m) at which the probability distribution was estimated was varied, with m = 256 (left), 512 (center) and 1024 (right). The error in the estimation of the probability distribution was measured for each well as the Kolmogorov-Smirnov statistic between the actual cell fluorescence values and the estimated probability distribution. [file 12859_2015_636_MOESM6_ESM.pdf]

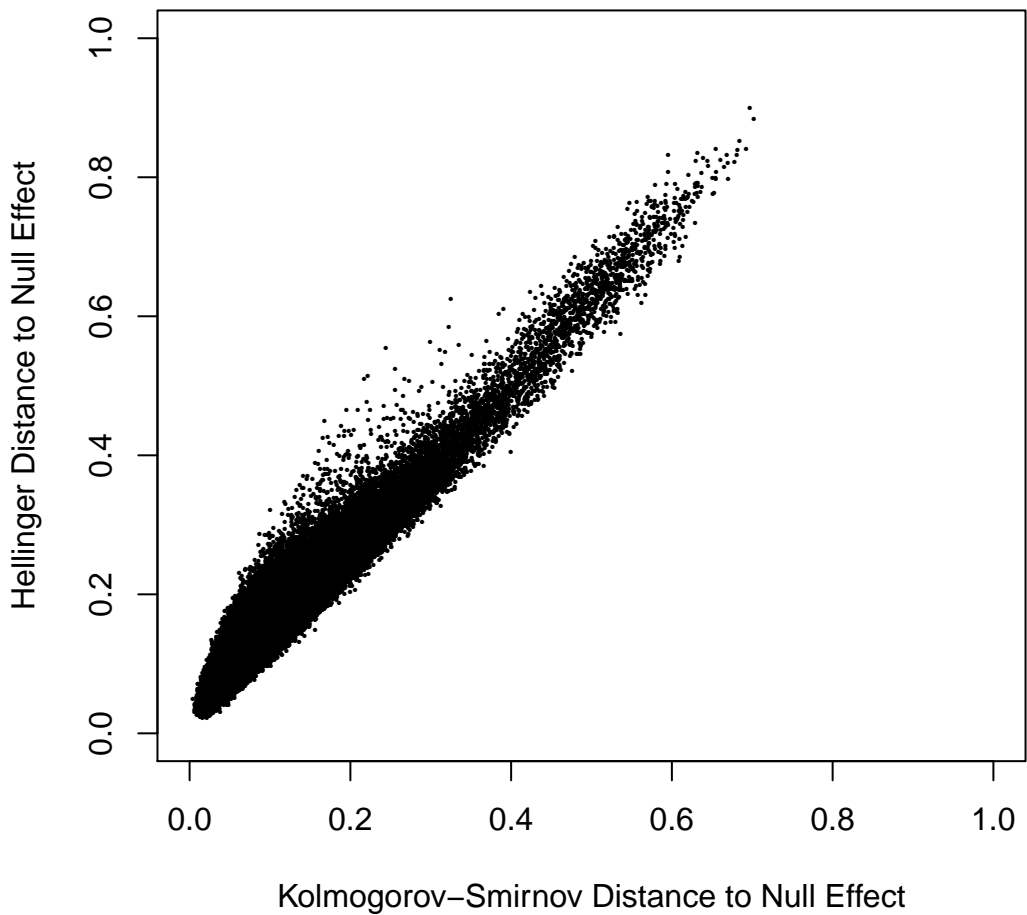

Supplement: Additional file 8: Figure S6. — Difference between Hellinger distance to null effect and Kolmogorov-Smirnov distance to null effect. The Hellinger distance of all treated wells in the screen to the null effect is plotted against the Kolmogorov-Smirnov distance between the same wells and the null effect. The distance metrics are very highly correlated. [file 12859_2015_636_MOESM8_ESM.pdf]

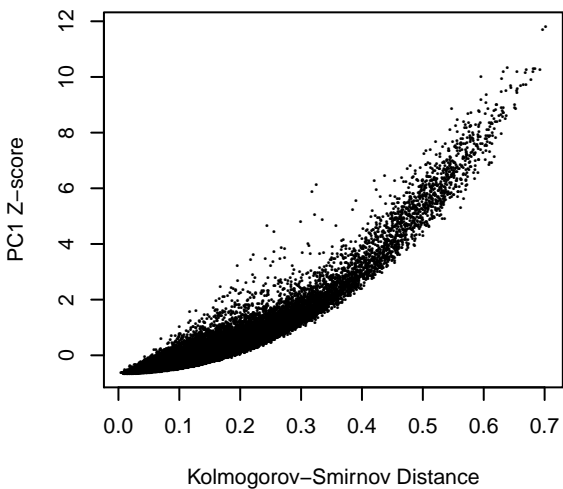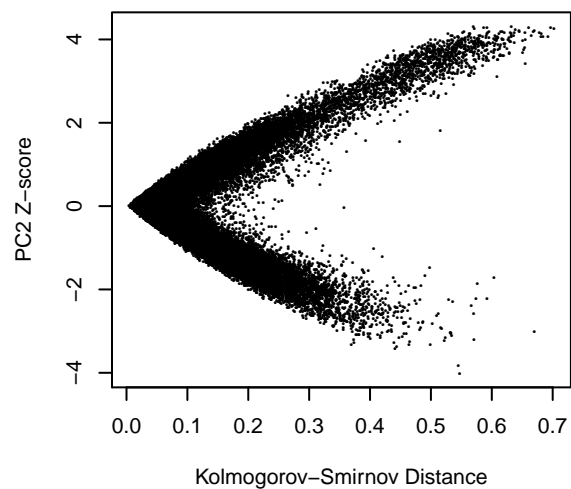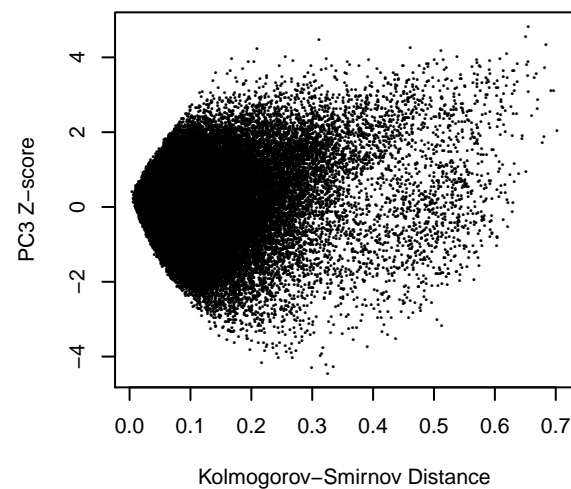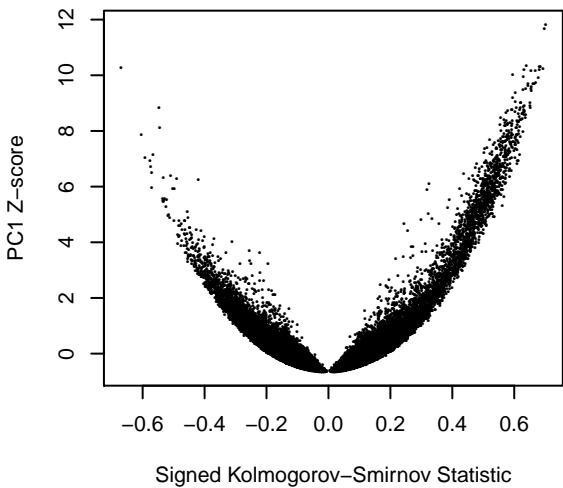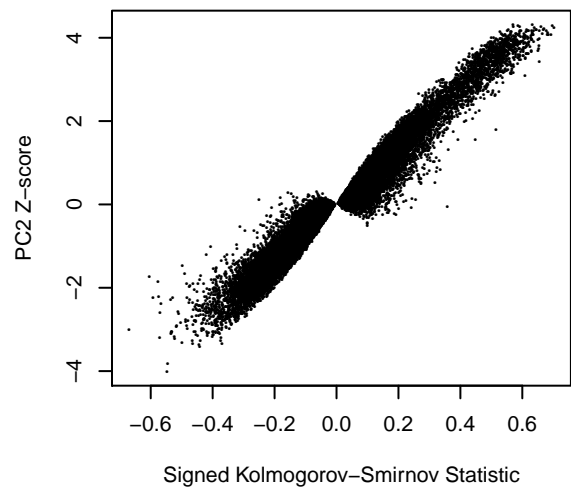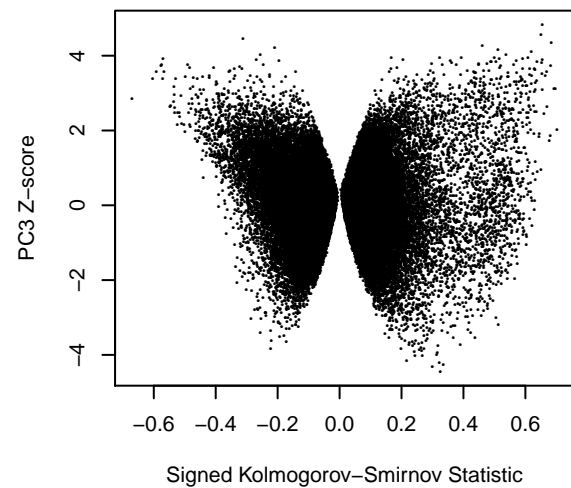

Supplement: Additional file 9: Figure S7. — Comparing the Kolmogorov-Smirnov statistic to null effect and the first three PC scores of the Hellinger distance embedding. The Kolmogorov-Smirnov distance (always positive) (top) and the signed Kolmogorov-Smirnov statistic (possibly negative) (bottom) was computed for all wells in the screen and plotted against the first three PC scores of the Euclidean embedding of Hellinger distances. The third PC scores from the Hellinger distance embedding (right) are not substantially correlated with the Kolmogorov-Smirnov distances to null effect. [file 12859_2015_636_MOESM9_ESM.pdf]

### True Clustering

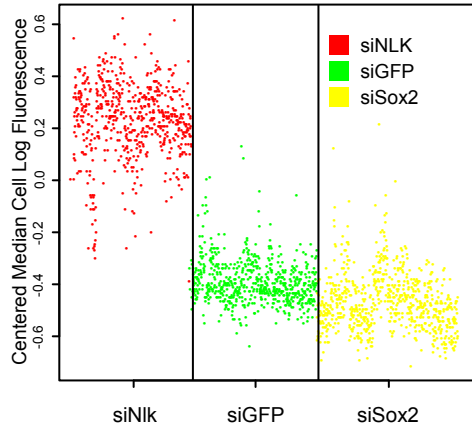

### Cluster by Distribution

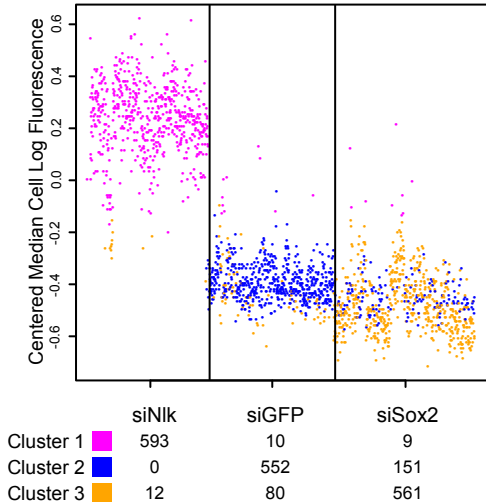

### Cluster by Gaussian Fit

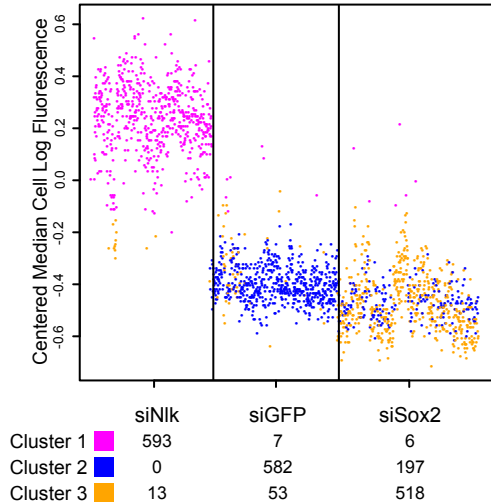

Supplement: Additional file 11: Figure S9. — Clustering controls using dual Gaussian mixture model. Median cell fluorescence for all control siRNA conditions that could be fit to convergence with a dual Gaussian mixture model. Values are ordered by category (siNlk, siGFP and siSox2) and separated by vertical bars. Clustering with 3 medoids by Hellinger distance between distributions (center) or by Hellinger distance between distribution fit to a dual Gaussian mixture model (right) is shown in magenta, blue and orange. Number of category conditions assigned to each cluster is shown as numbers beneath the category. [file 12859_2015_636_MOESM11_ESM.pdf]

## Cluster 1

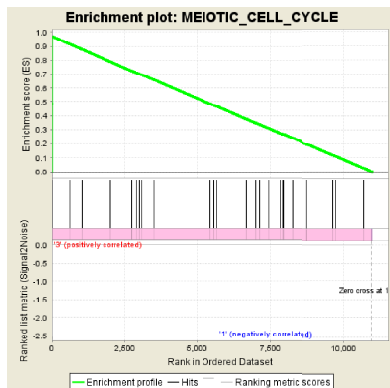

## Cluster 2

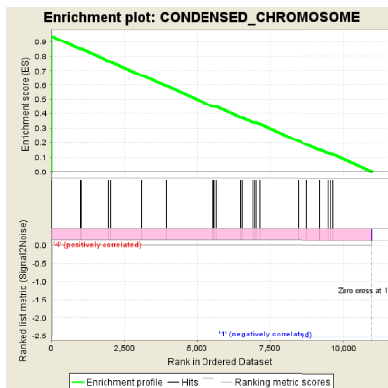

## Cluster 3

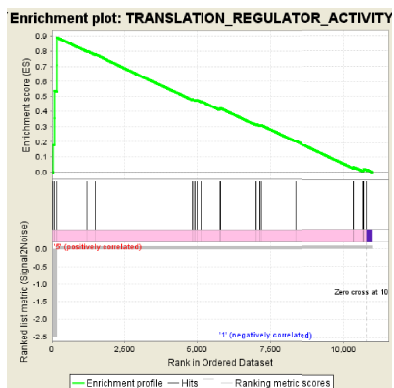

## Cluster 4

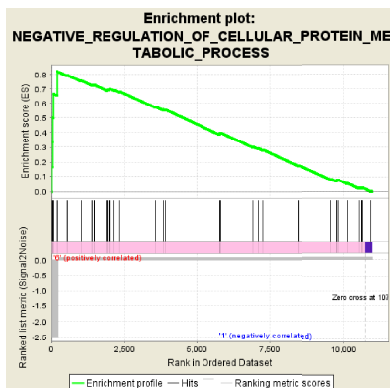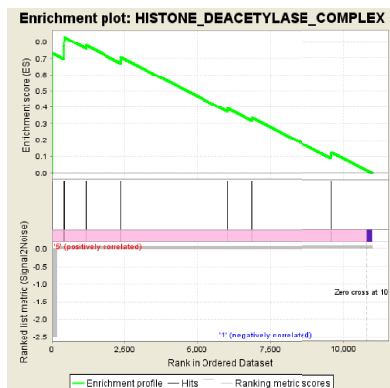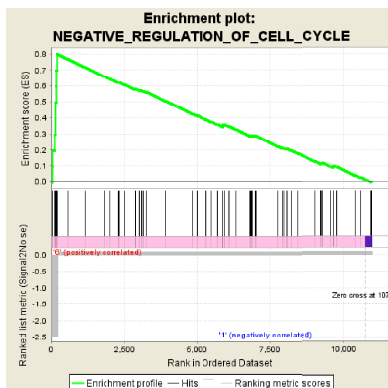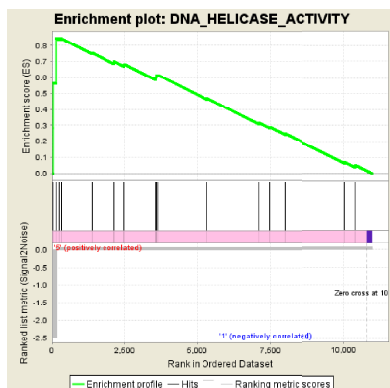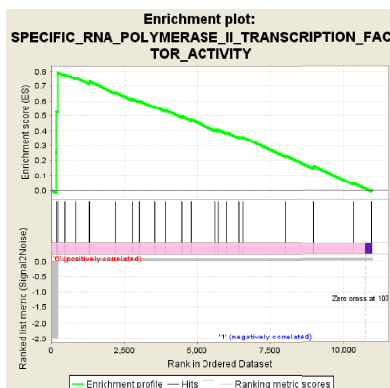

Supplement: Additional file 14: Table S3. — Gene sets enriched in the 4-medoid distribution clusters. Gene set enrichment analysis was performed on the unique mapped genes assigned to each outlier cluster distribution from a 4-medoid clustering. The tested motif gene sets in the Molecular Signatures Database are shown here. [file 12859_2015_636_MOESM14_ESM.pdf]
