# Supplementary material for: Distribution Analyzer, a methodology for identifying and clustering outlier conditions from single-cell distributions, and its application to a Nanog reporter RNAi screen
Source: BMC Bioinformatics. 2015 Jul 22;16:225. doi: 10.1186/s12859-015-0636-7 (PMC4511455; doi:10.1186/s12859-015-0636-7)
Supplement: Additional file 10: Figure S8. — Clustering controls using Kolmogorov Smirnov score to null effect. For each control well, the signed Kolmogorov-Smirnov distance was calculated between the cumulative distribution function of the cells in the well and the null-effect. Values are ordered by category (siNlk, siGFP and siSox2) and separated by vertical bars. Clustering with 3 medoids of the Kolmogorov-Smirnov scores (left) is compared to 3 medoid clustering by Hellinger distance (right). [file 12859_2015_636_MOESM10_ESM.pdf]

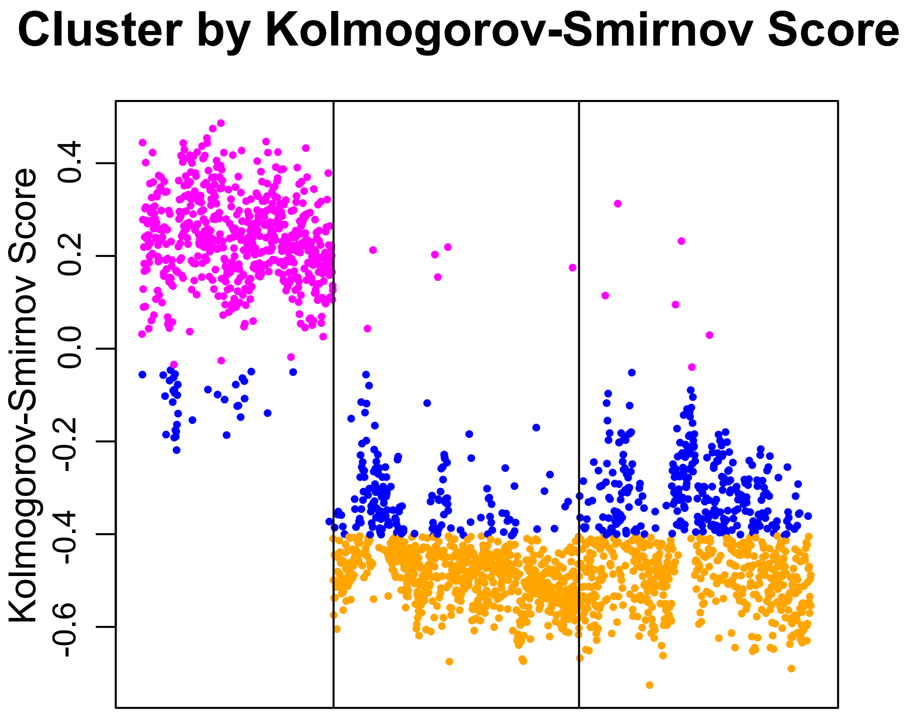

	siNlk	siGFP	siSox2
Cluster 1	593	6	6
Cluster 2	37	162	292
Cluster 3	0	641	468

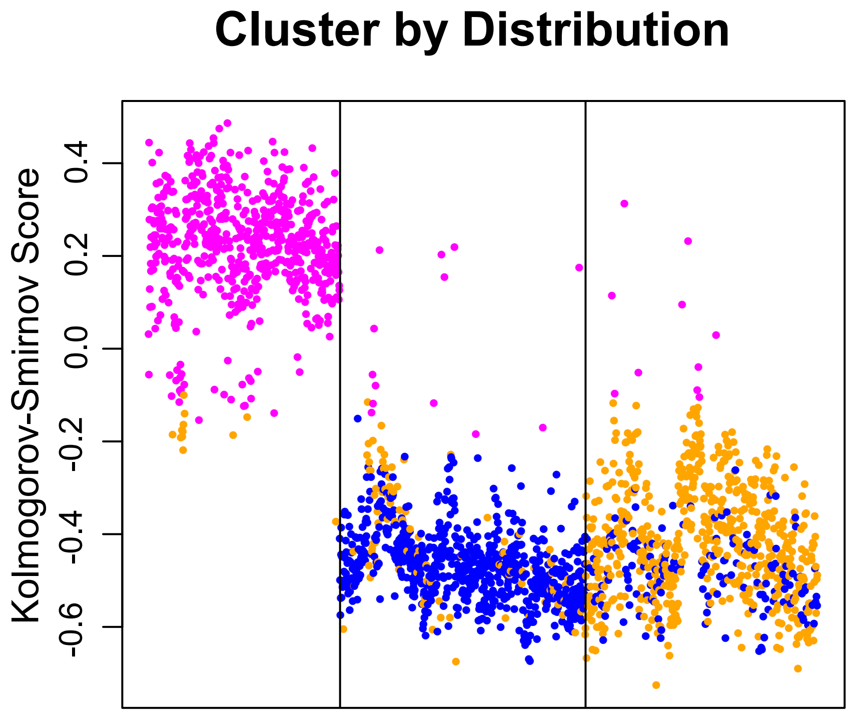

	siNlk	siGFP	siSox2
Cluster 1	618	13	10
Cluster 2	0	703	156
Cluster 3	12	93	600
